# Actomyosin contractility and a threshold of cadherin cell adhesion are required during tissue fusion

**DOI:** 10.1083/jcb.202503070

**Published:** 2025-11-13

**Authors:** Camilla S. Teng, Sarah W. Curtis, Grace C. Brewer, Elizabeth J. Leslie-Clarkson, Jeffrey O. Bush

**Affiliations:** 1 Department of Cell and Tissue Biology, University of California San Francisco, San Francisco, CA, USA; 2 Program in Craniofacial Biology, University of California San Francisco, San Francisco, CA, USA; 3 Institute for Human Genetics, University of California San Francisco, San Francisco, CA, USA; 4 Eli and Edythe Broad Center of Regeneration Medicine and Stem Cell Research, University of California San Francisco, San Francisco, CA, USA; 5 Department of Human Genetics, Emory University School of Medicine, Atlanta, GA, USA

## Abstract

Tissue fusion is integral to mammalian morphogenesis, and its failure is a significant cause of structural anomalies, yet the underlying cellular mechanisms are incompletely understood. We examine cellular drivers of upper lip fusion in the mammalian embryo by establishing a live-imaging modality, revealing specific enrichment of F-actin that propagates in multicellular cables anchored at the fusion site. Actomyosin contractility drives lip fusion, and its pharmacological or genetic attenuation results in failed fusion and cleft lip. Generating a series of mice deficient in specific p120-catenin molecular functions, we reveal that p120-catenin binding to RhoA and Kaiso is dispensable during mammalian development, while stabilization of cadherins is crucial. Through generating an allelic series of new compound P-cadherin/E-cadherin mouse mutations disrupting combined cadherin levels, we unveil an elevated cadherin cell adhesion threshold requirement specific to upper lip fusion. Finally, we identify *CDH3* variants in individuals with cleft lip, supporting the relevance of this mechanism in human tissue fusion.

## Teaser

Teng et al. investigate the cellular basis for tissue fusion during mammalian lip formation. They demonstrate that actomyosin contractility drives fusion, creating a threshold requirement for cadherin-based cell adhesion. These results also define p120-catenin molecular functions during development and implicate this mechanism in cleft lip, a common structural anomaly.

## Introduction

Tissue fusion, wherein two distinct embryonic primordia come together to become one continuous structure, is a common theme in embryonic development across the animal kingdom and requires an assemblage of intricate cell behaviors coordinated in space and time. For example, in *Drosophila* dorsal closure, the flanking epithelia are pulled together and fused to cover an epidermal gap on the dorsal side (Franke et al., 2005), and in chordate spinal cord formation, closure of the neural tube requires fusion of the dorsal neuroepithelium (Galea et al., 2017). Multiple tissue fusion events are required for normal mammalian craniofacial development, and their disruption is a meaningful contributor to congenital disease (Ray and Niswander, 2012).

**Figure 1.**
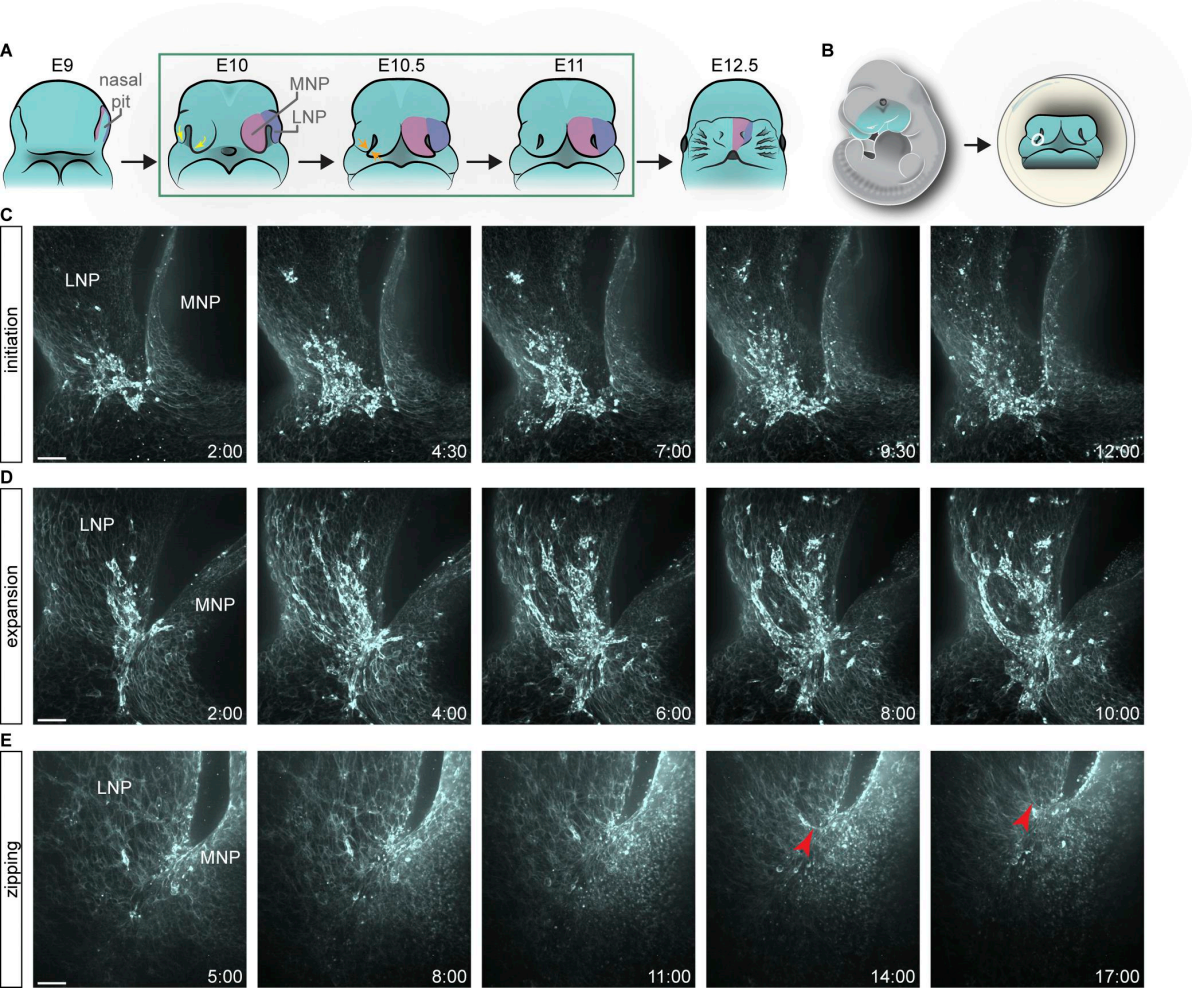
**Live imaging reveals actin dynamics at the fusion site. (A)** Schematic depicting lip morphogenesis and imaging approach. Yellow arrows indicate prominences coming together; orange arrows indicate the site of tissue fusion. The green box indicates stages of imaging. **(B)** Schematic of the tissue included in the explant. The white circle indicates the imaged region. **(C)** Time-lapse stills of labeled F-actin at 6ts reveal initiation of actin enrichment at what will become the fusion junction (*n* = 10). **(D)** Time-lapse stills of labeled F-actin at 10ts reveal expansion of actin to multicellular cables (*n* = 3). **(E)** Time-lapse stills of labeled F-actin at 16ts reveal zipping of nasal processes (*n* = 3). Red arrowhead indicates actin cable. MNP, medial nasal process; LNP, lateral nasal process. Time reads as hh:mm. Scale bars, 50 μm.

Failed tissue fusion results in cleft lip, a common congenital anomaly occurring with or without cleft palate affecting roughly one in 700 live births worldwide (Dixon et al., 2011). Static views and histological sections of mammalian embryos during development have provided a gross understanding of the embryology of upper lip formation in mammals (Jiang et al., 2006; Cox, 2004) (Fig. 1 A). Upper lip morphogenesis is a multistep process that initiates with the formation of the medial and lateral nasal processes (MNP and LNP, respectively). The MNP and LNP are joined at their caudal ends, where they also meet with the maxillary process (MXP), at a point sometimes referred to as the lambdoidal junction (Tamarin and Boyde, 1977; Ferretti et al., 2011; Losa et al., 2018). Fusion is initiated between the MNP and LNP in a posterior-to-anterior direction in a zippering-like process that brings the epithelia of the two structures into contact followed by regression of the intervening epithelium to fully unite them (Trasler, 1968; Gaare and Langman, 1977a; Gaare and Langman, 1977b). All of this occurs concomitantly to overall tissue expansion, making the development of the upper lip a three-dimensionally complex and dynamic tissue morphogenesis. Static cytological and immunohistochemical studies in combination with studies of tissue fusion in other contexts have enabled postulation of some of the cellular behaviors that may be involved in lip development. Cell cycle arrest and high levels of apoptosis within the epithelium of the MNP/LNP junction are correlated with successful upper lip fusion (Jiang et al., 2006; Ferretti et al., 2011; Losa et al., 2018; Ji et al., 2020; Qu et al., 2025, *Preprint*). The cellular drivers of this process have not been directly functionally assessed, however, and the origins of cell-based forces powering this remarkable tissue fusion remain largely mysterious.

Generally, actomyosin contractility driven by non-muscle myosin II (NMII) is thought to generate mechanical force in mammalian cells and is key for cell movement and tissue shape changes during embryonic development (Vicente-Manzanares et al., 2009). Single-nucleotide variants in *MYH9*, encoding non-muscle myosin IIA, are associated with non-syndromic cleft lip with or without cleft palate in humans (Martinelli et al., 2007; Birnbaum et al., 2009; Chiquet et al., 2009). Cell–cell adhesion mediated by cadherins at adherens junctions connects to the actomyosin cytoskeleton and contributes to mechanical coupling during morphogenesis (Mège and Ishiyama, 2017; Heisenberg and Bellaïche, 2013). Notably, in humans, numerous mutations in genes encoding proteins of the adherens junction complex, including core components such as E-CADHERIN (*CDH1*) and P120-CATENIN (*CTNND1*), have been implicated in causing cleft lip as part of blepharocheilodontic syndrome and non-syndromic cleft lip (Kievit et al., 2018; Cox et al., 2018). A recent study found that *CTNND1* was recurrently mutated in a study of 841 orofacial cleft (OFC) cases, making it the most common genetic cause of cleft lip in that population (Diaz Perez et al., 2023). P120-catenin is notable for its function as part of adherens complexes, regulating cadherin levels by suppressing their degradation by the proteasome (Ireton et al., 2002; Davis et al., 2003). Additionally, p120-catenin can regulate the cytoskeleton by interacting with Rho GTPases (Anastasiadis et al., 2000; Grosheva et al., 2001; Anastasiadis and Reynolds, 2001), can interact with the transcription factor Kaiso to regulate transcription, and can both facilitate and antagonize Wnt signaling (Daniel and Reynolds, 1999; Kelly et al., 2004; Spring et al., 2005; Hernández-Martínez et al., 2019). To date, there is only limited delineation of the context-specific cellular functions of p120-catenin deployed *in vivo* in mammalian development.

In this study, we interrogate cellular mechanisms of lip formation, focusing particularly on actomyosin contractility, apoptosis, and cell–cell adhesion. We use a combination of live imaging to examine the local dynamics and function of actomyosin contractility and a series of new genetic alleles in mice to dissect the *in vivo* molecular functions of p120-catenin and cadherin-mediated adhesion in this process. Our studies reveal locally enriched F-actin during lip fusion and indicate that actomyosin forces are required to pull embryonic prominences together. This fusion morphogenesis demands an elevated cadherin adhesion threshold satisfied through combined E-cadherin and P-cadherin levels.

## Results

### F-actin is dynamically elevated at the fusion site, and actomyosin contractility is essential for proper lip development

**Figure S1.**
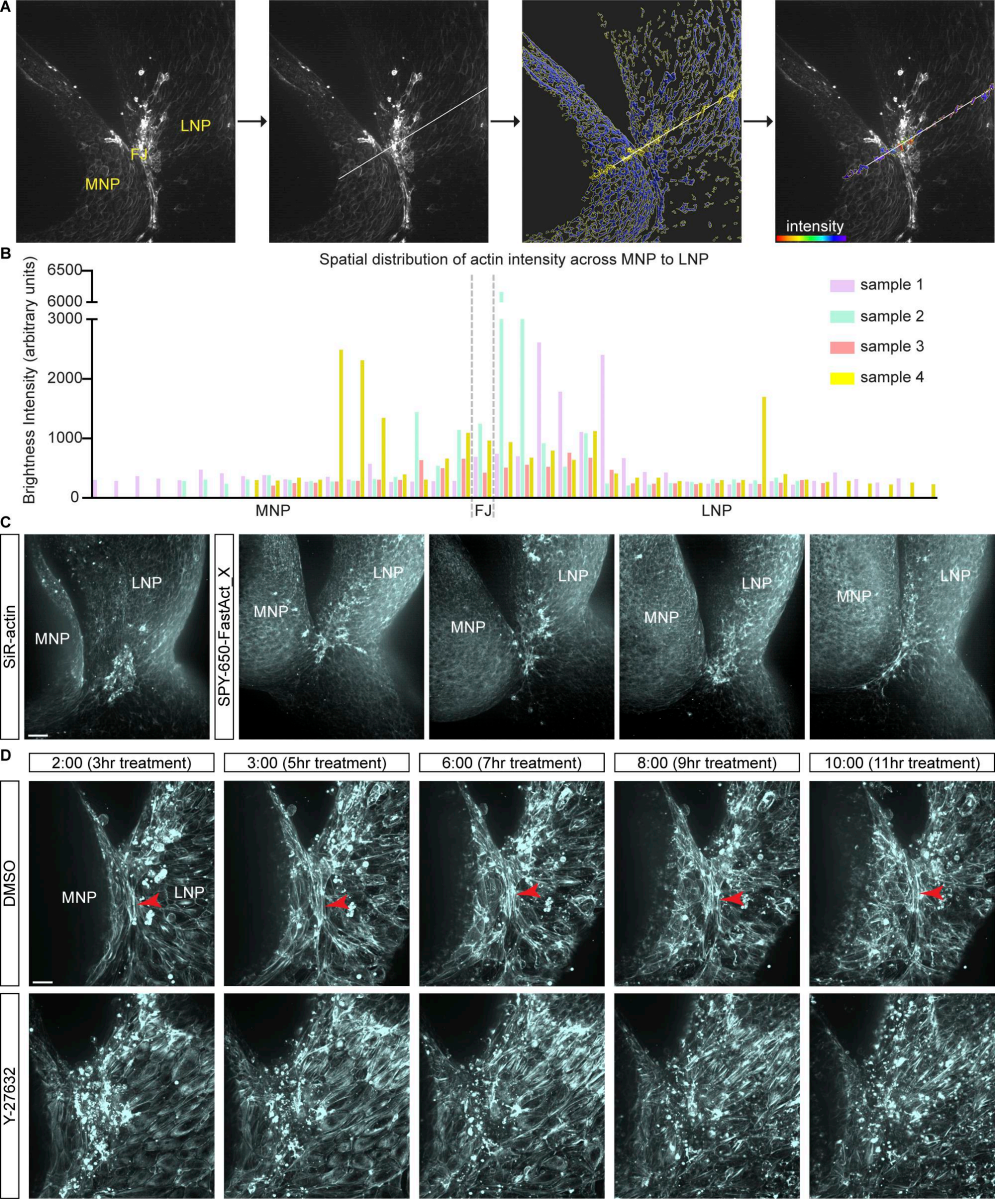
**Assessing F-actin distribution and role of actomyosin contractility in wild-type embryos. (A)** Quantifying distribution of actin across nasal processes during fusion. A line drawn across fusing nasal processes is used to select representative actin surfaces in creating an intensity profile. **(B)** Graph shows spatial distribution of surface intensities (y axis) across tissue (x axis), peaking broadly at the junction and junction-adjacent LNP, in four individual samples (9–11ts). FJ, fusion junction; MNP, medial nasal process; LNP, lateral nasal process. Scale bar, 50 μm. **(C)** Examples from four different wild-type embryos (7–8ts) stained with SPY-650-FastAct_X show actin enrichment at fusion region similar to SiR-Actin–stained embryo (7ts). MNP, medial nasal process; LNP, lateral nasal process. Scale bar, 40 μm. **(D)** Actomyosin contractility is required for lip fusion. Time-lapse stills of F-actin in 10ts samples treated with DMSO or Y-27632. Treatment began 1 h prior to imaging. Multicellular actin cables (red arrowhead) form at the fusion junction in DMSO-treated samples (*n* = 3) but did not form in 2/3 Y-27632–treated samples. MNP, medial nasal process; LNP, lateral nasal process. Time reads as hh:mm. Scale bar, 20 μm.

The dynamic cellular processes underlying upper lip development remain unknown. As forces generated by the actomyosin cytoskeleton are a major driver of morphogenesis generally (Vicente-Manzanares et al., 2009), we developed a strategy for the live imaging of actomyosin dynamics during upper lip fusion in mouse embryos. We dissected the embryonic midface, comprising the MNP, LNP, MXP, and eyes, and cultured it in the presence of SiR-Actin, a fluorogenic probe for live cells based on F-actin binding jasplakinolide conjugated to a bright and photostable silicon-rhodamine fluorophore (Lukinavičius et al., 2014) (Fig. 1 B). We live-imaged wild-type embryos at multiple stages defined by the number of tail somites (ts), to capture the entire process of lip fusion. We identified three phases of actin dynamics, which we refer to as “initiation,” “expansion,” and “zipping.” At the initiation phase, roughly 6ts or embryonic day (E) 10.0, wild-type embryos exhibit dramatic accumulation of F-actin in the epithelium at the MNP and LNP junction with a particular enrichment in the LNP epithelium, which was highlighted by quantification of F-actin signal intensities (Fig. 1 C; Video 1; and Fig. S1, A and B). While the overall pattern of actin enrichment varied meaningfully from embryo to embryo, we consistently observed enrichment in the fusion junction–adjacent LNP. This pattern was also observed using a fluorogenic FastAct_X probe, which binds to but does not stabilize F-actin (Nasufovic et al., 2025, *Preprint*) (Fig. S1 C); all subsequent experiments were performed with SiR-Actin only. Later, at roughly 10ts or E10.5, F-actin remains enriched at the fusing seam and expands laterally through the epithelium of the MNP and LNP in long multicellular cables appearing to pull together the two nasal processes initiating fusion, leading us to consider this the “expansion phase” (Fig. 1 D and Video 2). Finally, at roughly 16ts or E11.0, when a substantial amount of fusion has already occurred, we detected supracellular actin cables that continue to zipper up the nasal processes to shorten the nasal slit, which we named the “zipping phase” (Fig. 1 E and Video 3). These results from using our novel live-imaging approach unveil a dynamic actin network that is patterned at the fusing nasal processes.

**Video 1.** **Initiation of actin enrichment.** Time-lapse movie of labeled F-actin at 6ts reveals initiation of actin enrichment at what will become the fusion junction. Time reads as hh:mm:ss. Scale bar, 50 μm. Related to Fig. 1.

**Video 2.** **Expansion of actin to multicellular cables.** Time-lapse movie of labeled F-actin at 10ts reveals expansion of actin to multicellular cables. Time reads as hh:mm:ss. Scale bar, 50 μm. Related to Fig. 1.

**Video 3.** **Actin zipping of nasal processes.** Time-lapse movie of labeled F-actin at 16ts reveals zipping of nasal processes. Red arrowhead indicates actin cable. Time reads as hh:mm:ss. Scale bar, 50 μm. Related to Fig. 1.

**Figure 2.**
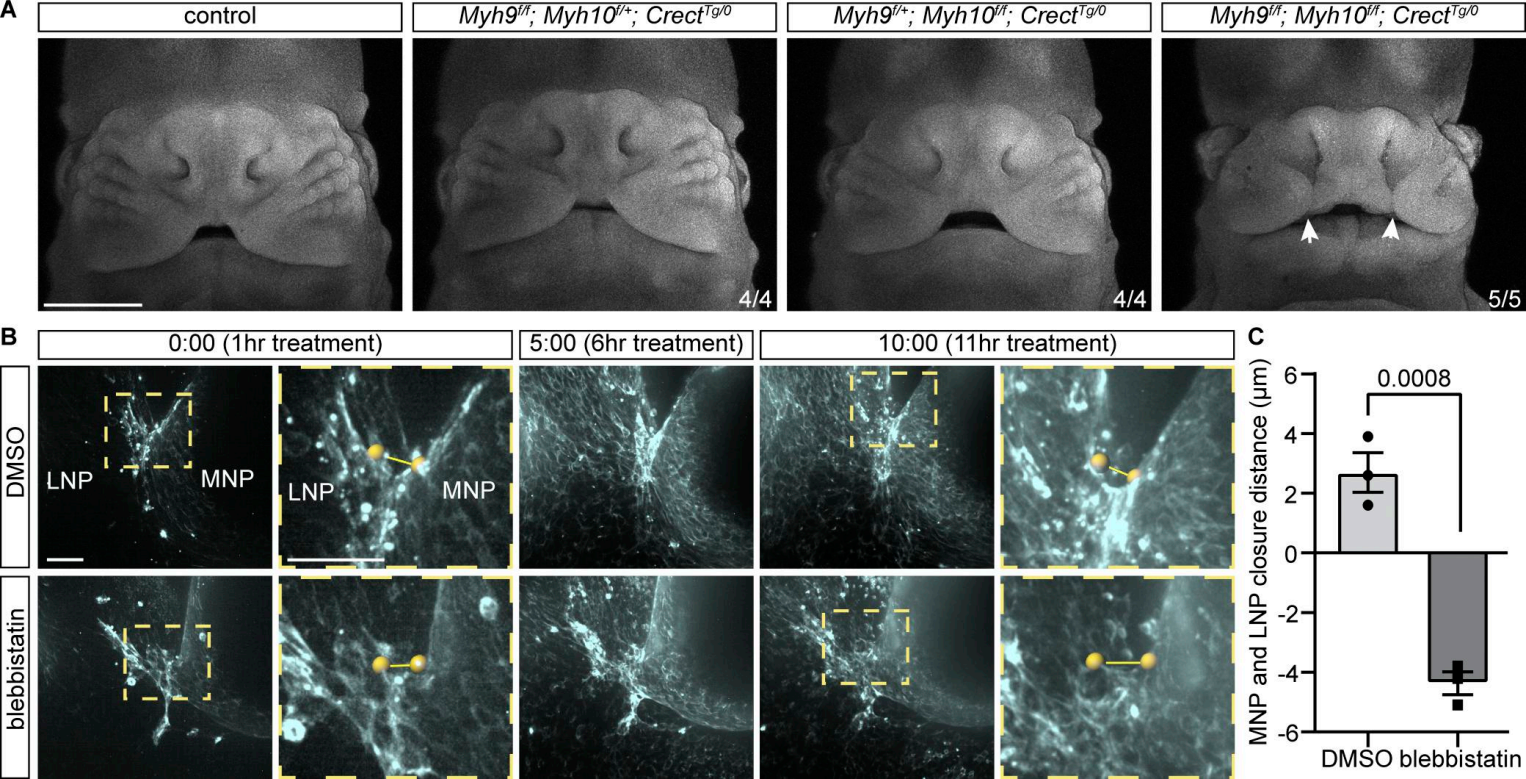
**Actomyosin contractility is required for lip fusion. (A)** Bilateral cleft lip (white arrows) is detected in E12.5 *Myh9*^*f/f*^; *Myh10*^*f/f*^; *Crect*^*Tg/0*^ embryos lacking NMII function in the epithelium versus control, *Myh9*^*f/f*^; *Myh10*^*f/+*^; *Crect*^*Tg/0*^, and *Myh9*^*f/+*^; *Myh10*^*f/f*^; *Crect*^*Tg/0*^ embryos. Scale bar, 1 mm. Images were adjusted to gamma 1.3 to achieve more even brightness. **(B)** Time-lapse stills of F-actin in 9ts samples treated with DMSO (*n* = 3) or blebbistatin (*n* = 3). Treatment began 1 h prior to imaging. Yellow spheres and lines indicate distance measurement between MNP and LNP. MNP, medial nasal process; LNP, lateral nasal process. Time reads as hh:mm. Scale bars, 50 μm. **(C)** Blebbistatin-treated samples had less closure between nasal processes than DMSO-treated samples. Dots represent individual samples. Error bars represent the SEM. Data were compared using two-tailed Student’s *t* test.

We next tested if and how actomyosin contractility is crucial to lip fusion. As the enriched actin observed in live imaging appeared localized to the epithelium, we reasoned that an epithelial Cre driver would be appropriate to ablate non-muscle myosin function during lip fusion. Therefore, we conditionally deleted *Myh9* and *Myh10*, which encode the non-muscle myosin heavy chain IIA (NMHCIIA) and NMHCIIB isoforms, respectively, using the *Crect* craniofacial ectoderm Cre. Whole-mount nuclear staining of embryos at E12.5 revealed that while *Myh9*^*f/f*^; *Myh10*^*f/+*^; *Crect* and *Myh9*^*f/+*^; *Myh10*^*f/f*^; *Crect* mutants are comparable to controls, *Myh9*^*f/f*^; *Myh10*^*f/f*^; *Crect* double mutants exhibited bilateral cleft lip (Fig. 2 A). This result undoubtedly reveals a role of actomyosin contractility in proper lip formation; however, the extent to which it specifically drives fusion as opposed to other processes was unclear. To evaluate the impact of actomyosin contractility perturbation on the fusion process per se, we took advantage of our live imaging setup and commonly used pharmacological inhibitors. We added blebbistatin, a myosin II inhibitor, or dimethyl sulfoxide (DMSO) as a control condition to the culture media of tissue explants from 9ts wild-type embryos prior to imaging. Over the course of 10 h in controls, F-actin accumulated at the fusion junction as described above and fusion was readily observed (Fig. 2, B and C; and Video 4). While there was initial F-actin enrichment at the fusion junction in the blebbistatin-treated condition, the propagation of F-actin was dissipated and the MNP and LNP were separated (Fig. 2, B and C; and Video 5). Treatment with Y-27632, a ROCK inhibitor, also results in the lack of multicellular actin cables characteristic of the fusion junction and overtly appeared to disrupt fusion similar to blebbistatin (Fig. S1 D; and Videos 6 and 7). Thus, perturbation of actomyosin contractility results in stagnant fusion of nasal processes and ultimately cleft lip, revealing a critical role of actomyosin contractility in bringing the MNP and LNP together in the fusion process.

**Video 4.** **Tissue fusion proceeds in control DMSO treatment.** Time-lapse movie of F-actin at 9ts treated with DMSO. Treatment began 1 h prior to imaging. Time reads as hh:mm:ss. Scale bar, 50 μm. Related to Fig. 2.

**Video 5.** **Stagnant tissue fusion with disrupted actomyosin contractility.** Time-lapse movie of F-actin at 9ts treated with blebbistatin. Treatment began 1 h prior to imaging. Time reads as hh:mm:ss. Scale bar, 50 μm. Related to Fig. 2.

**Video 6.** **Tissue fusion in control DMSO treatment.** Time-lapse movie of F-actin at 10ts treated with DMSO. Treatment began 1 h prior to imaging. Time reads as hh:mm:ss. Scale bar, 20 μm. Related to Fig. S1.

**Video 7.** **Disrupted tissue fusion with disrupted actomyosin contractility.** Time-lapse movie of F-actin at 10ts treated with Y-27632. Treatment began 1 h prior to imaging. Time reads as hh:mm:ss. Scale bar, 20 μm. Related to Fig. S1.

### Global epithelial loss of p120-catenin results in localized epithelial disruption at the fusion junction

**Figure S2.**
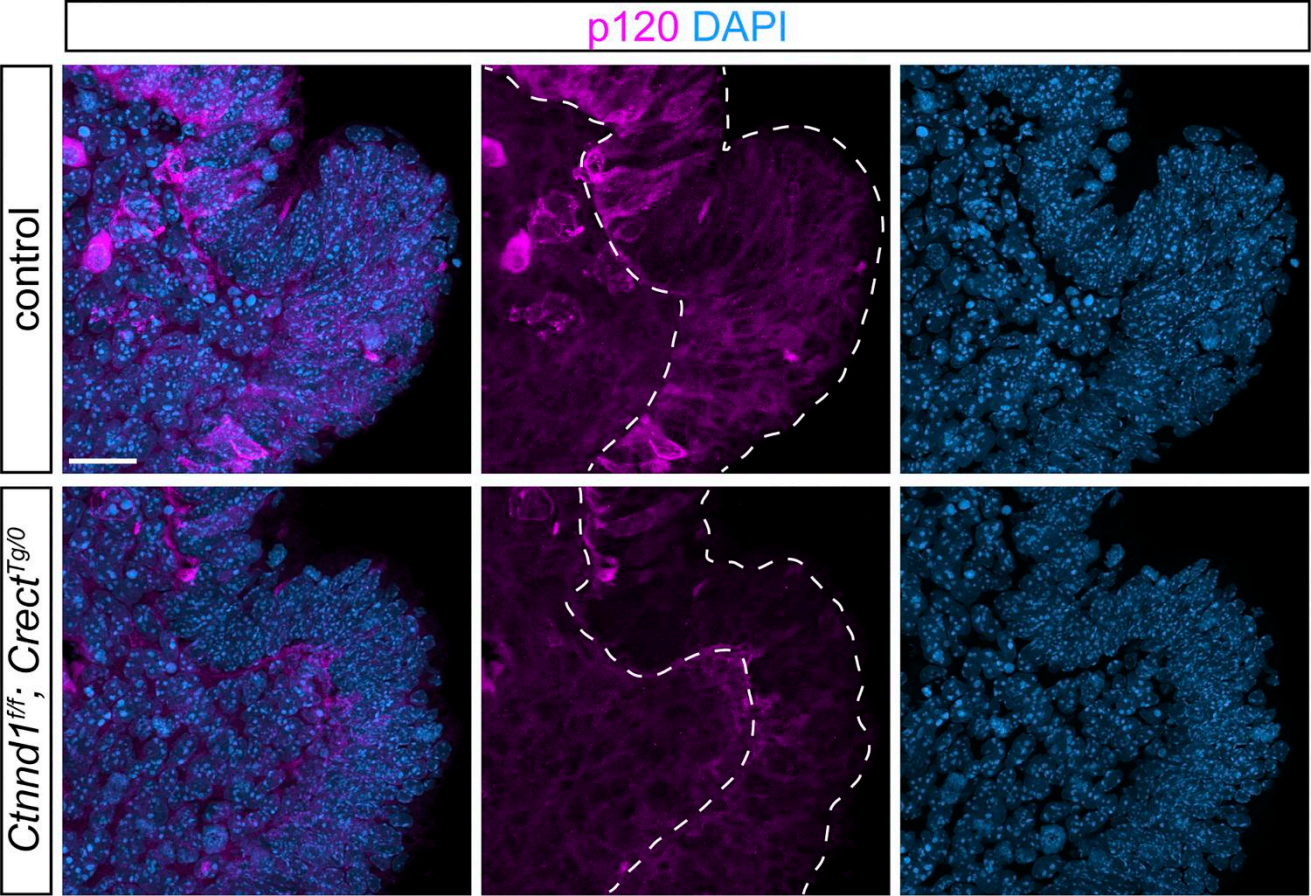
**Validation of epithelial-specific p120-catenin mutant.** Immunofluorescence staining on sections of the prefusion junction at 5–7ts reveals decreased epithelial p120-catenin in *Ctnnd1*^*f/f*^; *Crect*^*Tg/0*^ mutants (*n* = 3) compared with controls (*n* = 3). Scale bar, 20 μm.

**Figure 3.**
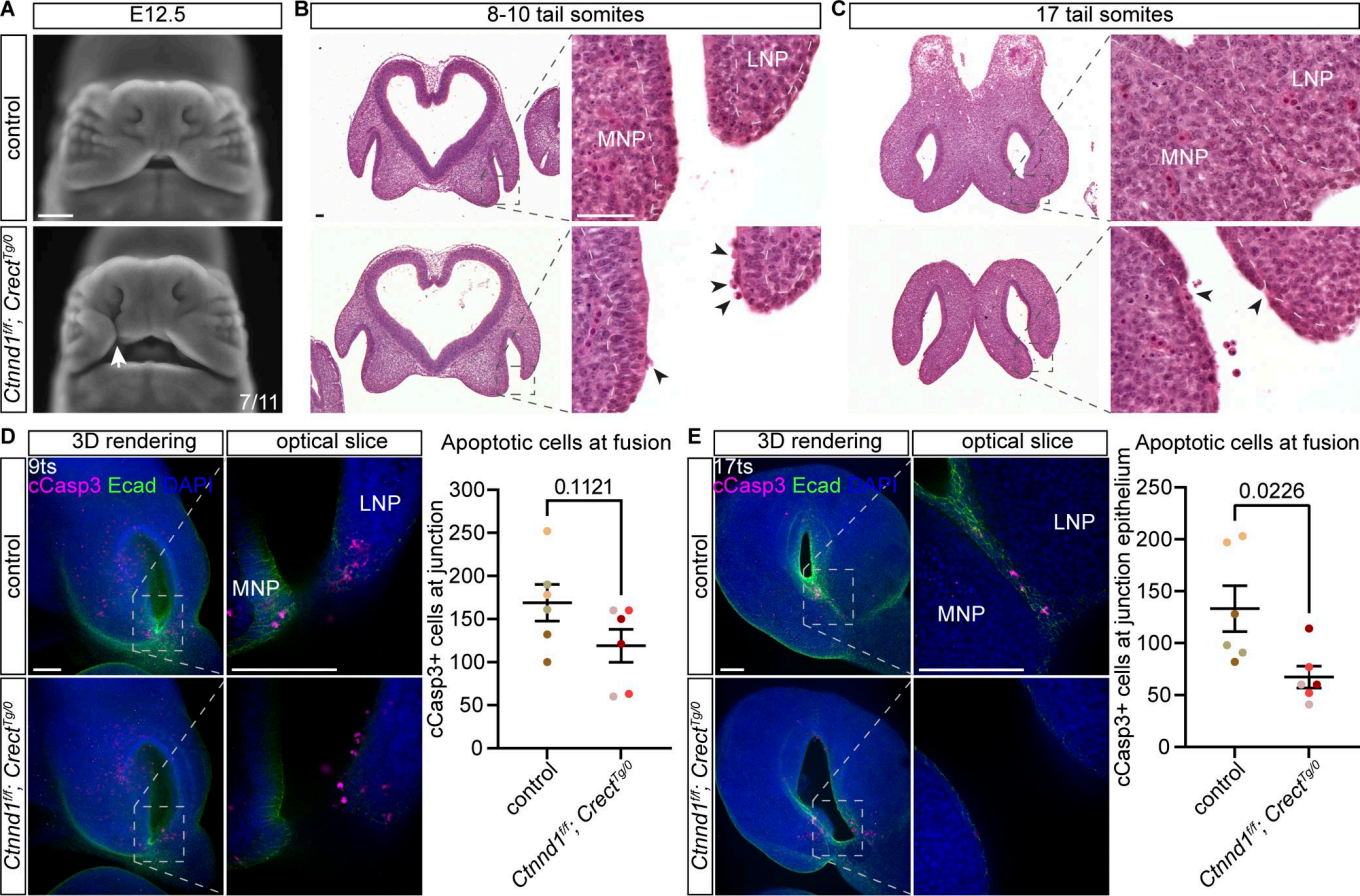
**Epithelial loss of p120-catenin results in localized epithelial disruption and decreased apoptosis at the fusion junction. (A)** Cleft lip (white arrow) is detected in 7/11 of E12.5 *Ctnnd1*^*f/f*^; *Crect*^*Tg/0*^ embryos versus controls. Scale bar, 500 μm. **(B and C)** Histological sections of control and *Ctnnd1*^*f/f*^; *Crect*^*Tg/0*^ embryos at 8–10ts (B) (*n* = 3 each) and 16–22ts (C) (*n* = 2 for controls, *n* = 3 for mutants). Black arrowheads point to disrupted epithelium. Scale bars, 50 μm. **(D and E)** Quantification of apoptosis in the prefusion (D) and fusion (E) region in whole-mount cCasp3-immunostained control and *Ctnnd1*^*f/f*^; *Crect*^*Tg/0*^ embryos (*n* = 3 each for each stage). Dot colors in graphs represent two sides of a single sample. MNP, medial nasal process; LNP, lateral nasal process. Scale bars, 100 μm. Data were compared using two-tailed Student’s *t* tests.

Actomyosin-generated cellular forces are transduced by mechanical coupling by cell–cell adherens junctions. We therefore next sought to dissect the cellular requirements of adherens junctions for fusion during lip morphogenesis. Others have previously noted that epithelial loss of p120-catenin results in cleft lip in mice (Cox et al., 2018), and we recapitulated this in embryos with conditional deletion of *Ctnnd1* driven by the craniofacial ectoderm Cre, *Crect*, which we confirmed mediated loss of p120-catenin specifically within the epithelium in *Ctnnd1*^*f/f*^; *Crect*^*Tg/0*^ mutant embryos (Fig. S2). The majority of whole-mount nuclear–stained E12.5 *Ctnnd1*^*f/f*^; *Crect*^*Tg/0*^ mutant embryos exhibited cleft lip, compared with littermate controls that displayed completed fusion and a normal upper lip (Fig. 3 A and Table S1). Histological analysis of *Ctnnd1*^*f/f*^; *Crect*^*Tg/0*^ mutant embryos revealed epithelial disorganization at 9ts (E10.5) that was strikingly limited to the zone where we observed actomyosin contractility enrichment when the MNP and LNP were first making contact in controls (Fig. 3 B). Whereas controls formed an extended epithelial seam by 17ts (E11), *Ctnnd1*^*f/f*^; *Crect*^*Tg/0*^ mutant embryos failed to do so, leaving a gap between the MNP and LNP with disorganized epithelium specifically within fusing regions where actomyosin contractility would normally be elevated (Fig. 3 C). Notably, the epithelium remains intact and apparently normal in regions away from the actomyosin-enriched fusion zone.

### Epithelial apoptosis does not account for cleft lip

Previous research on upper lip fusion has shown enrichment of apoptosis in the fusing epithelial seam, also known as the nasal fin (Jiang et al., 2006; Gaare and Langman, 1977b; Song et al., 2009). Analysis of cleaved caspase-3 by immunostaining in control embryos revealed apoptosis in the prefusion epithelium of the MNP and LNP at 9ts (Fig. 3 D), and also in the fusing nasal fin at 17ts (Fig. 3 E). Apoptosis appeared reduced in *Ctnnd1*^*f/f*^; *Crect*^*Tg/0*^ mutants at both 9ts and 17ts, but when quantified, this difference was only statistically significant at 17ts when fusion had failed, with cleaved caspase 3(+) cells remaining adjacent to but not a part of the epithelium (Fig. 3, D and E).

**Figure 4.**
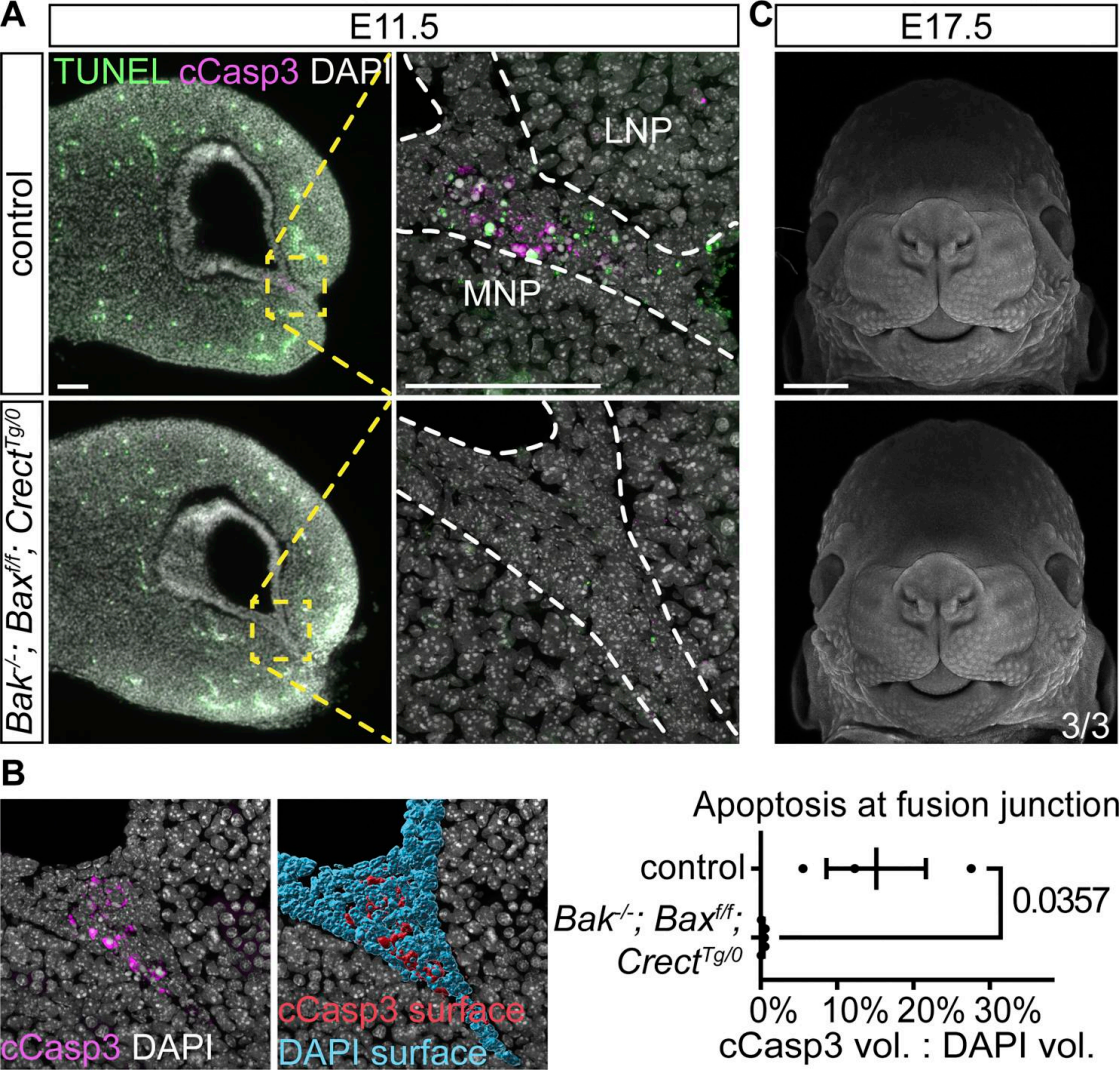
**Epithelial loss of apoptosis does not disrupt lip development. (A)** Immunofluorescence and TUNEL assay indicate the absence of cell death in the epithelium of *Bak*^*−/−*^; *Bax*^*f/f*^; *Crect*^*Tg/0*^ embryos (*n* = 5) versus controls (*n* = 3) at E11.5. MNP, medial nasal process; LNP, lateral nasal process. Scale bars, 70 μm. **(B)** In control example shown, surfaces were generated from cCasp3+ and DAPI signal at the fusion site. The percentage of cCasp3+ apoptosis in the fusion junction of *Bak*^*−/−*^; *Bax*^*f/f*^; *Crect*^*Tg/0*^ embryos (*n* = 5) is significantly lower than in control embryos (*n* = 3). Each dot indicates one sample. Error bars represent the SEM. Data were compared using a two-tailed Mann–Whitney *t* test. **(C)***Bak*^*−/−*^; *Bax*^*f/f*^; *Crect*^*Tg/0*^ E17.5 embryonic faces appear indistinguishable from controls. Scale bar, 1 mm.

While apoptosis is thought to be a major cellular mechanism for lip fusion, its requirement has not been fully established. Thus, we interrogated whether apoptosis is necessary for proper lip fusion by abrogating intrinsic apoptosis in the epithelium through disruption of the pro-apoptotic regulators Bax and Bak (Westphal et al., 2011) in *Bak*^*−/−*^; *Bax*^*f/f*^; *Crect*^*Tg/0*^ mutants. We observed near-complete loss of all cell death at the nasal fin at E11.5 through cleaved caspase-3 staining and TUNEL assay (Fig. 4 A), which was confirmed by quantification of cleaved caspase-3 surface volumes (Fig. 4 B), indicating that apoptosis in this context occurs through the intrinsic apoptotic pathway. At E17.5, whole-mount nuclear–stained *Bak*^*−/−*^; *Bax*^*f/f*^; *Crect*^*Tg/0*^ mutants appear indistinguishable from control littermates (Fig. 4 C), suggesting that apoptosis in the epithelium is dispensable for successful lip fusion, though it may still contribute in combination with other cell behaviors. This result is also congruent with our recent finding that cell death is not needed for mouse secondary palate fusion, despite the prevailing idea that apoptosis is required for resolving the epithelial seam (Teng et al., 2022).

### p120-catenin–cadherin interaction is crucial for upper lip fusion

**Figure 5.**
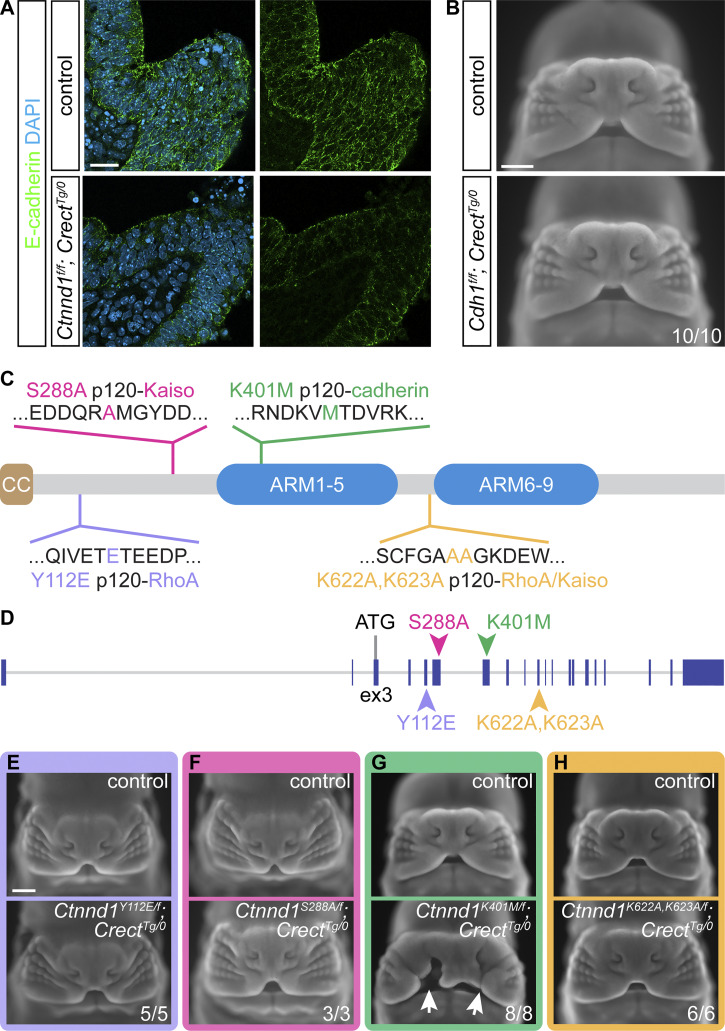
**Dissecting the molecular functions of p120-catenin *in vivo*. (A)** Optical slices of immunostained tissue sections show decreased E-cadherin in *Ctnnd1*^*f/f*^; *Crect*^*Tg/0*^ mutants (*n* = 2) compared with controls (*n* = 2) at 10ts. Scale bar, 20 μm. **(B)***Cdh1*^*f/f*^; *Crect*^*Tg/0*^ embryo faces appear similar to controls at E12.5. Scale bar, 500 μm. **(C)** Schematic of p120-catenin protein illustrating the location of amino acid substitutions. CC, coiled-coil domain; ARM, armadillo domain. **(D)** Schematic of *Ctnnd1* illustrating the location of corresponding point mutations. **(E–H)** Four *Ctnnd1* point mutation alleles examined at E12.5 compared with controls. Only *Ctnnd1*^*K401M/f*^; *Crect*^*Tg/0*^ mutants (G) (8/8) exhibit cleft lip (arrows). Scale bar, 500 μm.

**Figure 6.**
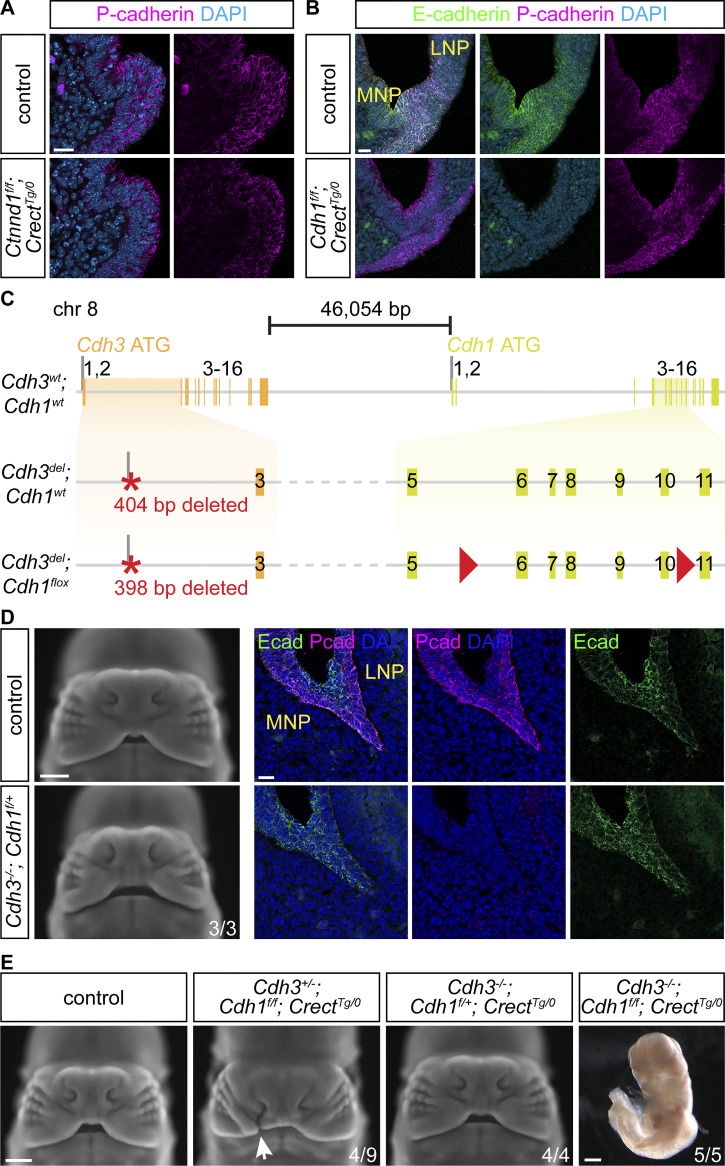
**E-cadherin and P-cadherin comprise a critical cadherin threshold at the fusion site. (A)** Optical slice of immunostained tissue sections shows decreased P-cadherin in *Ctnnd1*^*f/f*^; *Crect*^*Tg/0*^ mutants (*n* = 3) compared with controls (*n* = 3) at 5–7ts. Scale bar, 20 μm. **(B)** Immunofluorescence on sections of the prefusion junction detects decreased E-cadherin but not P-cadherin in 10ts *Cdh1*^*f/f*^; *Crect*^*Tg/0*^ mutants (*n* = 5) compared with controls (*n* = 5). MNP, medial nasal process; LNP, lateral nasal process. Scale bar, 20 μm. **(C)** Schematic of *Cdh1*/*Cdh3* loci depicting *Cdh3* mutations with linked *Cdh1* alleles. Red triangles, loxP sites. **(D)***Cdh3*^*−/−*^; *Cdh1*^*f/+*^ embryos appear similar to controls at E12.5. Scale bar, 500 μm. Immunofluorescence on sections of the fusion junction detects decreased P-cadherin but not E-cadherin in 20ts *Cdh3*^*−/−*^; *Cdh1*^*f/f*^ mutants (*n* = 4) compared with control (*n* = 4). MNP, medial nasal process; LNP, lateral nasal process. Scale bars, 20 μm. **(E)** Cleft lip (white arrow) was detected in 4/9 E12.5 *Cdh3*^*+/−*^; *Cdh1*^*f/f*^; *Crect*^*Tg/0*^ but not *Cdh3*^*−/−*^; *Cdh1*^*f/+*^; *Crect*^*Tg/0*^ embryos. *Cdh3*^*−/−*^; *Cdh1*^*f/f*^; *Crect*^*Tg/0*^ embryos at E10.5 exhibit dramatic dysmorphology. Scale bars, 500 μm.

Given the prominent role of p120-catenin in adherens junctions, and recent findings that show mutations in *CDH1* in human cleft lip, we anticipated that E-cadherin, a core component of epithelial adherens complexes, would be required for lip fusion. Immunostaining cryosections of 10ts embryos for E-cadherin revealed high expression and localization to the epithelial cell membrane of control embryos, whereas *Ctnnd1*^*f/f*^; *Crect*^*Tg/0*^ mutants displayed a substantial decrease of E-cadherin in the epithelium (Fig. 5 A), consistent with the role of p120-catenin in preventing E-cadherin endocytosis and degradation. Epithelial-specific disruption of *Cdh1* led to E-cadherin loss (Fig. 6 B), but, astonishingly, did not result in cleft lip, and E12.5 *Cdh1*^*f/f*^; *Crect*^*Tg/0*^ mutant embryos were indistinguishable from littermate controls (Fig. 5 B).

**Figure S3.**
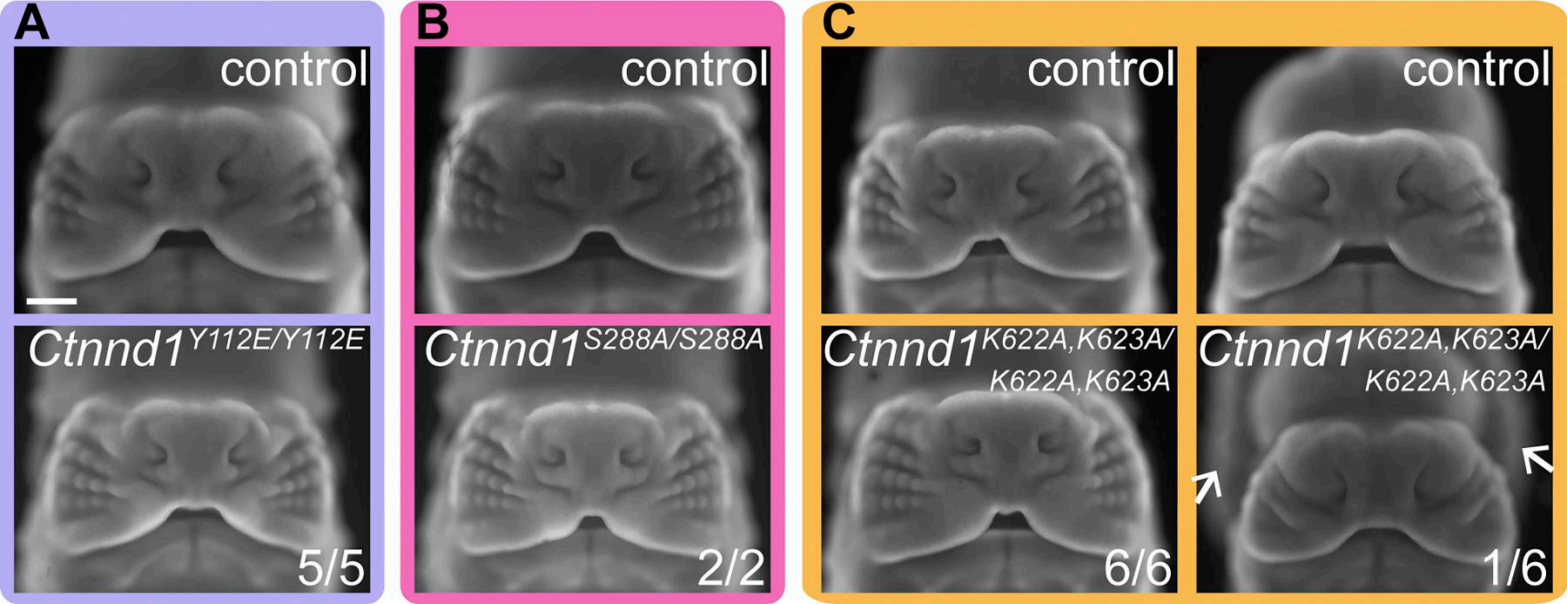
**Homozygosity of p120-catenin variants. (A–C)** *Ctnnd1* ^*Y112E/Y112E*^ embryos (A), *Ctnnd1*^*S288A/S288A*^ embryos (B), and *Ctnnd1*^*K622A,K623A/K622A,K623A*^ mutants (C) at E12.5 do not exhibit cleft lip. Arrows indicate exencephaly, which was observed in 1/6 embryos examined. Scale bar, 500 μm.

Thus, we investigated which functions of p120-catenin are necessary for lip development. P120-catenin is a multifunctional protein that interacts with numerous other proteins and can regulate the cytoskeleton through RhoA association and facilitate WNT signaling through repression of the Kaiso transcriptional repressor, in addition to its role in stabilizing cadherins. As such, we sought to molecularly dissect its association with Kaiso, RhoA, and cadherins to determine the function of p120-catenin in lip development. We generated four new mouse lines harboring amino acid substitutions previously established in cell culture studies to affect specific functions of p120-catenin. These amino acid substitutions in the p120-catenin protein include (1) Y112E that results in decreased p120-catenin/RhoA interaction (Castaño et al., 2007); (2) S288A that results in decreased p120-catenin/Kaiso interaction (Zhang et al., 2011); (3) K622A,K623A that results in decreased p120-catenin interaction with both RhoA and Kaiso (Anastasiadis et al., 2000; Kelly et al., 2004); and (4) K401M that results in decreased p120-catenin/cadherin interactions (Yu et al., 2016; Ishiyama et al., 2010) (Fig. 5 C). To generate *Ctnnd1* alleles that harbor genetic edits that would produce the above amino acid changes, we utilized the CRISPR/Cas9-based technique known as improved-Genome editing via Oviductal Nucleic Acids Delivery (*i*-GONAD) (Takahashi et al., 2015; Ohtsuka et al., 2018) (Fig. 5 D). To interrogate these functions of p120-catenin specifically in lip fusion, we generated compound heterozygous mice harboring one *Ctnnd1* floxed allele with the other allele carrying one of these four function-ablating point mutations and examined them at E12.5 by whole-mount nuclear staining. Notably, *Ctnnd1*^*Y112E/f*^; *Crect*^*Tg/0*^ mutants, *Ctnnd1*^*S288A/f*^; *Crect*^*Tg/0*^ mutants, and *Ctnnd1*^*K622A,K623A/f*^; *Crect*^*Tg/0*^ mutants did not exhibit cleft lip (Fig. 5, E–G), indicating that RhoA and Kaiso binding are not required for p120-catenin function in this context. Cleft lip was only observed in *Ctnnd1*^*K401M/f*^; *Crect*^*Tg/0*^ mutants (Fig. 5 H), pointing to a crucial role of p120-catenin/cadherin interactions in the epithelium during lip development. Beyond craniofacial development, we were surprised to observe that E12.5 *Ctnnd1*^*Y112E/Y112E*^, *Ctnnd1*^*S288A/S288A*^, and *Ctnnd1*^*K622A,K623A/K622A,K623A*^ homozygous mutants also did not exhibit cleft lip and were viable and healthy as adult mice (Fig. S3, A–C), indicating that these interaction domains are not absolutely required for the many crucial *in vivo* functions of p120-catenin. Of note, we did detect low penetrant exencephaly in *Ctnnd1*^*K622A,K623A/K622A,K623A*^ mutants (Fig. S3 C), which is reminiscent of the phenotype caused by brain- and neural crest–specific loss of p120-catenin (Pieters et al., 2020). We did not recover any *Ctnnd1*^*K401M/K401M*^ embryos at E12.5, pointing to the importance of this interaction domain for general embryo development. Overall, these results indicate that p120-catenin binding to RhoA and Kaiso does not have a significant *in vivo* role in mammalian development, in contrast to the crucial p120-catenin regulation of cadherins. We therefore hypothesize that adherens junction regulation by p120-catenin is specifically vital for lip fusion.

### A critical cadherin threshold is required for lip fusion

**Figure S4.**
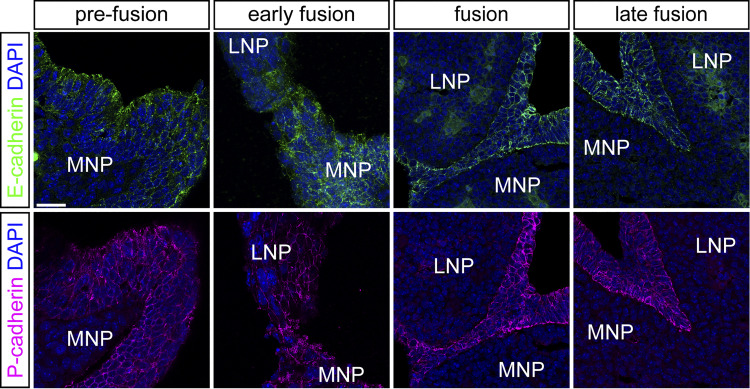
**E-cadherin and P-cadherin are present in the lip epithelium throughout the fusion process.** Immunofluorescence on control sections of the prefusion (6–8ts, *n* = 2 for E-cad, *n* = 3 for P-cad), early fusion (9–11ts, *n* = 5 for each), fusion (16–18ts, *n* = 2 for each), and late fusion (20–21ts, *n* = 4 for E-cad, *n* = 2 for P-cad) junction detects both E-cadherin and P-cadherin. MNP, medial nasal process; LNP, lateral nasal process. Scale bar, 20 μm.

As epithelial-specific loss of E-cadherin does not result in cleft lip (Fig. 5 B), we reasoned that a different cadherin may be involved in lip fusion. Although E-cadherin is the primary epithelial cadherin, P-cadherin is also present at the 6ts prefusion epithelium and somewhat lowered in *Ctnnd1*^*f/f*^; *Crect*^*Tg/0*^ mutants (Fig. 6 A). Further, we find both E and P-cadherin localized to the epithelium at 8–20ts, stages that span initiation, expansion, and zipping phases of lip fusion (Fig. S4). Intriguingly, P-cadherin has been shown to partially compensate for E-cadherin loss in some contexts (Tinkle et al., 2004). Immunostaining on cryosections shows P-cadherin persists in the fusion zone of 10ts *Cdh1*^*f/f*^; *Crect*^*Tg/0*^ mutants lacking E-cadherin (Fig. 6 B), hinting at a role of P-cadherin. Thus, to identify if P-cadherin is a key cadherin in lip fusion, we used *i*-GONAD to delete the first two exons of the P-cadherin–coding gene, *Cdh3*, generating a null mutant (Fig. 6 C). These *Cdh3*^*−/−*^ mutant mice do not exhibit cleft lip, and immunostaining shows E-cadherin persists in the fusion zone of 20ts *Cdh3*^*−/−*^ mutants lacking P-cadherin (Fig. 6 D). These mice are viable and fertile, consistent with previous findings that P-cadherin is not individually required for lip or general development (Radice et al., 1997).

We next sought to test whether the combined loss of E-cadherin and P-cadherin results in cleft lip. Complete loss of E-cadherin leads to early lethality at the blastocyst stage (Larue et al., 1994), and *Cdh1* and *Cdh3* are 46 kb apart, making generation of compound mutant embryos challenging through recombination during breeding (Hatta et al., 1991) (Fig. 6 C). Thus, we performed *i*-GONAD gene editing to generate a *Cdh3* loss-of-function mutation linked to the *Cdh1* floxed allele (Boussadia et al., 2002). *Cdh3*^*−/−*^; *Cdh1*^*f/f*^; *Crect*^*Tg/0*^ mutants exhibited catastrophic loss of cell adhesion and dramatic craniofacial phenotypes, dying by E9.5, thereby revealing their shared contributions to embryonic epithelial development, but also precluding our analysis of lip development (Fig. 6 E). To more finely reduce overall cadherin levels, we combined the linked *Cdh1* mutant; *Cdh3* floxed alleles with unlinked *Cdh1* and *Cdh3* alleles to generate an allelic series of cadherin loss. We discovered that a subset of *Cdh3*^*+/−*^; *Cdh1*^*f/f*^; *Crect*^*Tg/0*^ mutants exhibit cleft lip, while *Cdh3*^*−/−*^; *Cdh1*^*f/+*^; *Crect*^*Tg/0*^ mutants did not (Fig. 6 E and Table S1). Together, these data reveal a new role of P-cadherin in lip fusion and demonstrate that there is a critical threshold of cadherin-based cell adhesion needed for proper lip fusion.

### Actomyosin contractility and cell adhesion gene variants in human cleft lip

**Figure 7.**
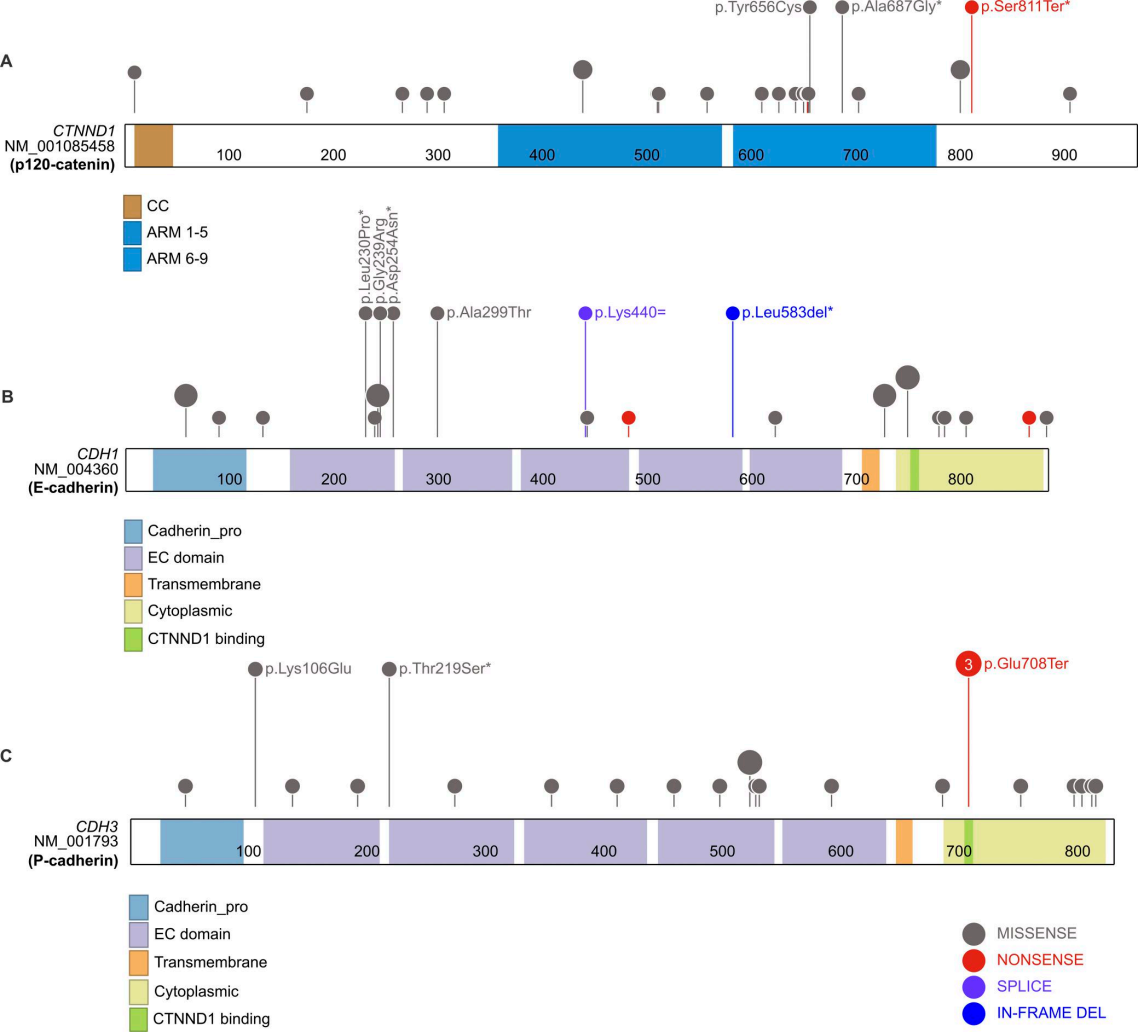
**Rare coding variants in cadherin and catenin pathway genes. (A–C)** Lollipop plots show the distribution of rare protein-altering variants in (A) *CTNND1*, (B) *CDH1*, and (C) *CDH3* identified in probands with cleft lip or cleft lip and palate. Protein domains are shown along the x axis with colors indicating distinct functional domains. CC, coiled-coil domain; ARM, armadillo domain; EC, extracellular cadherin. Each lollipop represents an individual variant; the size of the lollipop indicates how many times a variant was seen in our dataset. Variants with labels are *de novo* (marked with an asterisk), inherited from an affected parent, or classified as pathogenic in ClinVar. Variants include missense (gray lollipop), stop gain (red lollipop), splice region (purple lollipop), and in-frame indel (blue lollipop).

Previous studies indicate that *CDH1*, *CTNND1*, and *MYH9* gene disruption may contribute to cleft lip in humans. Though a *CDH3* homozygous truncating mutation has been previously implicated in ectodermal dysplasia, ectrodactyly, and macular dystrophy syndrome, these patients were not reported to exhibit orofacial clefting (Kjaer et al., 2005). We sought to examine *CDH3* and extend our understanding of the extent of involvement of actomyosin contractility and cell adhesion genes in cleft lip. In people with an OFC, we found 22 rare, protein-altering variants in *CTNND1*, 20 such variants in *CDH1*, 21 in *CDH3*, 28 in *MYH9*, and 24 in *MYH10* (Fig. 7 and Table S2). The combined annotation-dependent depletion (CADD) framework evaluates the potential pathogenicity of genetic variants (Kircher et al., 2014), and the majority of the variants we found (89.4%) have a CADD score >15 and thus would be predicted to be deleterious (Table S2). Seven variants are *de novo* (4 missense, 1 in-frame deletion, 1 nonsense, and 1 predicted splicing); three *de novo* variants (DNVs) were located in *CDH1*, two in *CTNND1*, one in *CDH3*, and one in *MYH10*. Seven of the inherited variants are inherited from a parent who also had an OFC. In *CDH1*, this included a missense variant and a synonymous variant predicted by SpliceAI to result in a loss of the splice donor site. In *CDH3*, this included a missense and nonsense variant. Notably, these *CDH1* and *CDH3* variants were absent from the exomes and genomes in the Genome Aggregation Database (gnomAD). The remaining variants inherited from affected parents were missense variants in *CTNND1* (1 variant) and *MYH9* (2 variants). All other rare variants were inherited from unaffected parents and may be considered variants of unknown significance, given the documented variable expressivity and incomplete penetrance among families with OFCs. When we tested for a burden of DNVs, we found that these five genes collectively had an enrichment of DNVs (P = 0.001), including an enrichment of missense (P = 0.008) and loss-of-function DNVs (P = 0.003) (Table S3). Most of this signal came from *CTNND1* (P = 0.006) and *CDH1* (P = 0.005), which were the only genes that also individually had a burden of DNVs (Table S3). However, even with those two genes removed, there was still a small enrichment of missense (0.04) and loss-of-function DNVs (P = 0.03) from variants in *CDH3*, *MYH9*, and *MYH10*. Of note, a *CDH3* variant found in three unrelated individuals with cleft lip is predicted to result in premature termination c.212G>T[p.Glu708Ter] within the p120-catenin interaction domain of P-cadherin (Fig. 7 C). For comparison, we selected 3 control genes (*MYH14*, *CDH5*, and *NKX2-1*) that we did not predict to be involved in craniofacial development based on known mouse phenotypes and expression patterns. Of these, only *MYH14* had a single DNV, which was not more than expected by chance (P = 0.22). In these control genes, there is no enrichment in missense variants (P = 0.32), protein-altering variants (P = 0.34), or all DNVs (P = 0.45) in this list. Together with other recently published human genetic findings (Cox et al., 2018; Diaz Perez et al., 2023; Alharatani et al., 2020), these data strongly indicate that disruption of the cell biological mechanisms we have uncovered here correlates with human cleft lip.

### Loss of adherens junctions results in cell rounding specifically in regions of high actomyosin contractility

**Figure 8.**
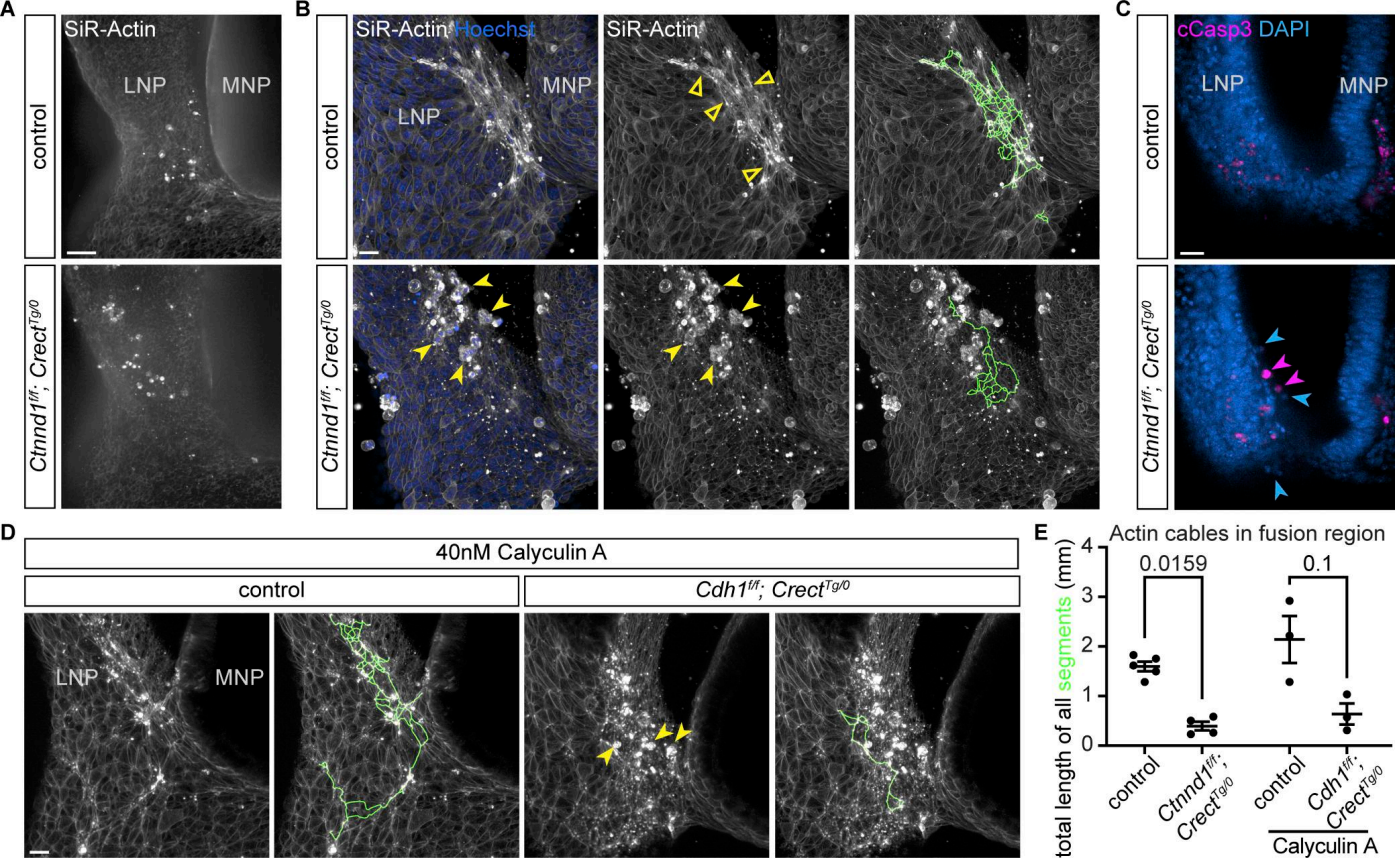
**Weakened adherens junctions are sensitized to heightened actomyosin forces. (A)** Labeling of F-actin at the fusion preinitiation stage (5–6ts) appears similar in controls (*n* = 3) and *Ctnnd1*^*f/f*^; *Crect*^*Tg/0*^ mutants (*n* = 3). MNP, medial nasal process; LNP, lateral nasal process. Scale bar, 50 μm. **(B)** At the initiation stage (8–11ts) in *Ctnnd1*^*f/f*^; *Crect*^*Tg/0*^ mutants (*n* = 3), rounded cells highly enriched in F-actin (yellow arrowheads) are found in the region that exhibits actin cables (open arrowheads) in controls (*n* = 5). Images were adjusted to gamma 2 to better visualize actin away from the fusion site. The green line represents Imaris-generated segments. Scale bar, 50 μm. **(C)** Optical slice of whole-mount–stained embryos (8ts) (*n* = 3 each). Rounded cells in *Ctnnd1*^*f/f*^; *Crect*^*Tg/0*^ mutants are often cCasp3^+^ (pink arrowheads) or cCasp3^-^ (blue arrowheads). Scale bar, 20 μm. **(D)** Actin labeling of control (*n* = 3) and *Cdh1*^*f/f*^; *Crect*^*Tg/0*^ mutants (*n* = 3) at the initiation stage (7–11ts) treated with calyculin A shows rounded cells (yellow arrowheads) in mutants. Gamma of images adjusted to 2. Green lines are Imaris-generated segments. Scale bar, 20 μm. **(E)** Lengths of Imaris-generated segments based on actin expression are less extensive in *Ctnnd1*^*f/f*^; *Crect*^*Tg/0*^ mutants and calyculin A–treated *Cdh1*^*f/f*^; *Crect*^*Tg/0*^ mutants than in respective controls. Each dot represents one sample. Error bars represent the SEM. Data were compared using two-tailed Mann–Whitney *t* tests.

To understand the interplay between actomyosin contractility and cell adhesion in lip fusion, we investigated the effects of adhesion loss on the dynamic and patterned actin network that we observed through live imaging of the fusing nasal processes. We dissected multiple stages of control and *Ctnnd1*^*f/f*^; *Crect*^*Tg/0*^ mutant embryos to capture different phases of the fusion process (Fig. 1 B). These live samples were labeled with SiR-Actin and/or Hoechst for F-actin and nuclei, respectively, and imaged. Prior to the initiation of fusion, actin enrichment was not yet observed, and control and mutant embryos were indistinguishable (Fig. 8 A). During the initiation phase, controls showed enriched actin organized into cables at and around the fusion site including the LNP as previously observed (Fig. 8 B). In *Ctnnd1*^*f/f*^; *Crect*^*Tg/0*^ mutants, however, F-actin accumulated at the fusion region lacked filamentous architecture, and quantification revealed significantly fewer organized cables in mutants (Fig. 8, B and E). Instead, in regions corresponding to those exhibiting F-actin multicellular cables in controls, we observed rounded cells enriched in F-actin that were detaching from the epithelium (Fig. 8 B). This is consistent with the spatially specific epithelial disorganization we observed through histological analysis of the mutants (Fig. 3, B and C). Some of these rounded cells exhibited cleaved caspase-3 staining and appeared to be separating from the epithelium, suggesting that these cells may be undergoing anoikis possibly as a consequence of loss of adhesion coupled with high cortical actomyosin contractility (Fig. 8 C). Taken together, we find that p120-catenin is not required for actin enrichment at the fusion site but is required for its organization into the multicellular actin cable patterns characteristic of active fusion.

**Figure S5.**
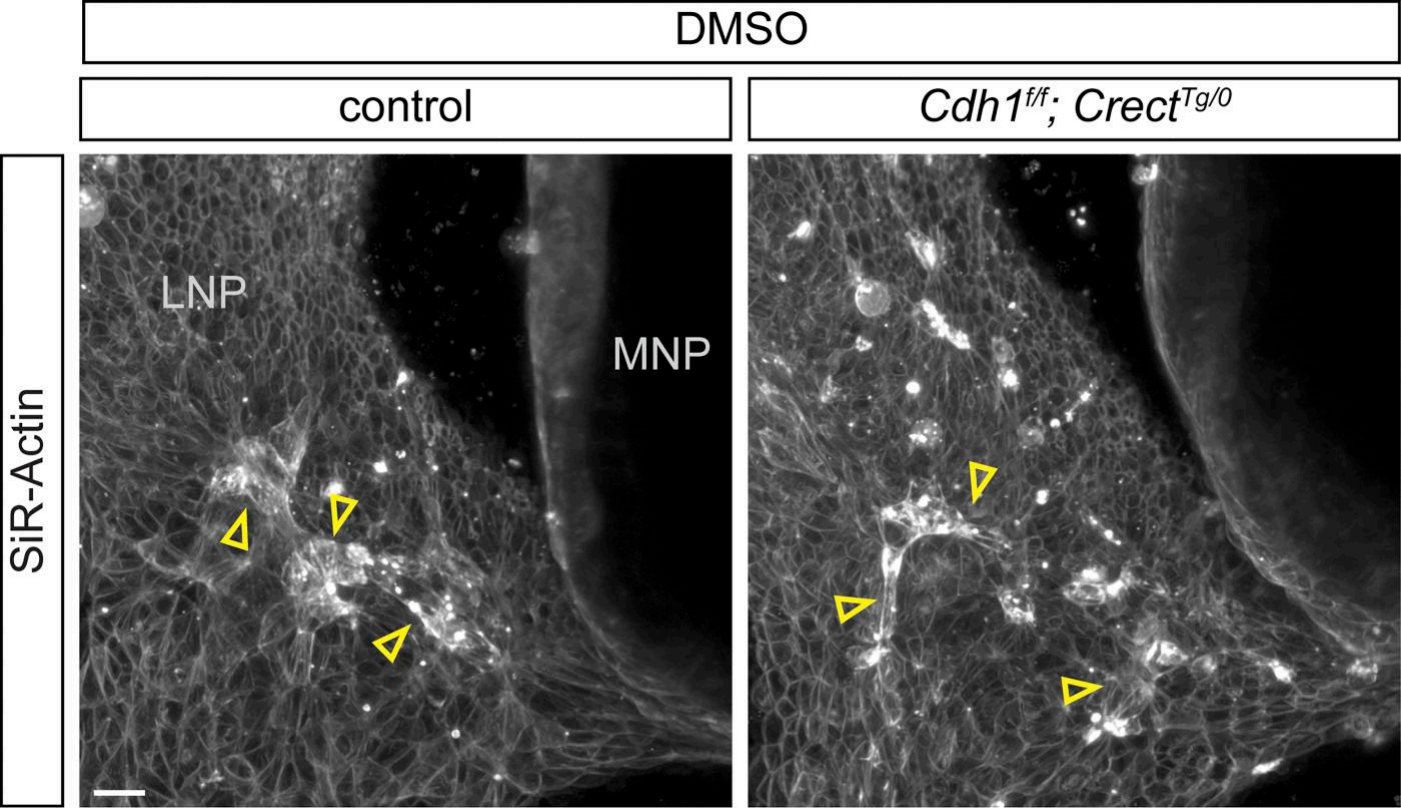
**Loss of E-cadherin does not affect F-actin enrichment in control conditions.** Labeling reveals actin enrichment at the initiation stage (8ts) (open arrowheads) of controls (*n* = 3) and *Cdh1*^*f/f*^; *Crect*^*Tg/0*^ mutants (*n* = 2) treated with DMSO. Gamma of images adjusted to 2. MNP, medial nasal process; LNP, lateral nasal process. Scale bar, 20 μm.

While *Cdh1*^*f/f*^; *Crect*^*Tg/0*^ mutants have lowered adherens junctions, the lack of cleft lip suggests that a critical adhesion threshold required during fusion is satisfied. To test this hypothesis, we challenged *Cdh1*^*f/f*^; *Crect*^*Tg/0*^ mutants by hyperactivating myosin contractility by treating with calyculin A, an inhibitor of myosin light chain phosphatase. Actin organization in tissue explants from 8ts *Cdh1*^*f/f*^; *Crect*^*Tg/0*^ mutants treated with DMSO and stained with SiR-Actin appeared comparable to littermate controls (Fig. S5). We then added calyculin A to tissue explants of 8ts control and *Cdh1*^*f/f*^; *Crect*^*Tg/0*^ mutant embryos 15 min prior to imaging. With this treatment, controls exhibited multicellular actin cables, while mutants had fewer and shorter cables and also rounded cells at the fusion region (Fig. 8, D and E), reminiscent of *Ctnnd1*^*f/f*^; *Crect*^*Tg/0*^ mutants. These results support the idea that a critical adhesion threshold must be met during lip fusion, possibly to withstand high actomyosin contractility forces.

## Discussion

Uncovering the cellular forces and mechanisms driving tissue fusion is essential to a comprehensive understanding of mammalian tissue morphogenesis. While principles of morphogenesis have been established in powerful non-mammalian model systems, our understanding of mammalian morphogenesis, and therefore the detailed cellular basis of human congenital anomalies, has lagged due to challenges intrinsic to live imaging of the viviparous mouse embryo. Here, we reveal dynamic cellular processes driving initiation of a mammalian tissue fusion through the establishment of a novel live-imaging platform for the upper lip. We demonstrate that F-actin is enriched in a highly localized pattern and that actomyosin-generated forces are crucial for drawing together embryonic prominences and zippering the upper lip. This is particularly notable in light of the fact that several studies have shown human cleft lip associated with mutations in *MYH9,* which encodes NMHCIIA (Martinelli et al., 2007; Birnbaum et al., 2009; Chiquet et al., 2009). Dorsal closure tissue fusion in *Drosophila*, ventral enclosure in *Caenorhabditis elegans* hypodermis, and spinal neural tube closure in mice are driven, at least in part, by the uniform contraction of a biomechanically coupled multicellular actomyosin purse-string, while neural tube closure in *Ciona robusta* occurs by sequential junctional actomyosin contraction that results in progression of a fusion zipper (Williams-Masson et al., 1997; Pyrgaki et al., 2010; Hashimoto et al., 2015; Wernike et al., 2016; Galea et al., 2017; Hayes and Solon, 2017; Hashimoto and Munro, 2019). We find that the zipping stage of upper lip fusion also exhibits purse-string-like actomyosin zippering to shorten the nasal slit, though the earlier initiation and expansion steps of upper lip fusion involve a unique pattern of F-actin enrichment that appears to pull the epithelia of the MNP and LNP together. This early and dramatic function of actomyosin contractility may be more akin to the requirement of actomyosin contractility in amnioserosa cell constriction that pulls the epidermal fusion fronts together in *Drosophila* dorsal closure. Our novel imaging approach establishes upper lip fusion as a system for future detailed studies to untangle the relative contributions of each of these actomyosin and other cellular mechanisms.

Through a battery of newly generated mouse mutants, we also reveal the detailed molecular requirements for p120-catenin during upper lip fusion. Previous studies have proposed that p120-catenin acts as a crucial scaffold protein that regulates numerous molecular functions (Anastasiadis et al., 2000; Anastasiadis and Reynolds, 2001; Daniel and Reynolds, 1999; Spring et al., 2005; Castaño et al., 2007; Zhang et al., 2011; Yu et al., 2016; Noren et al., 2000; McCrea and Park, 2007). P120-catenin can bind directly to RhoA in the cytoplasm and can act as a Rho guanine nucleotide dissociation inhibitor (RhoGDI) to inhibit RhoA activity (Anastasiadis et al., 2000; Castaño et al., 2007). Phosphorylation of the Tyr112 residue in p120-catenin prevents p120-catenin/RhoA binding, while mutation of two lysines, K622 and K623, disrupts the ability of p120-catenin to act as a RhoGDI (Anastasiadis et al., 2000; Castaño et al., 2007). In our study, mice harboring the Y112E phosphomimetic mutation exhibited no discernible phenotype, with adult *Ctnnd1*^*Y112E/Y112E*^ mice viable and healthy. This was also the case for most *Ctnnd1*^*K622A,K623A/K622A,K623A*^ homozygous mutant mice. While we have not confirmed that these mutations also disrupt p120-catenin/RhoA interactions *in vivo*, based on published work in cell culture, we interpret this to mean that binding of p120-catenin to RhoA and its ability to act as a GDI are both dispensable for normal development and viability in mice. These lysine mutations have also been shown to impact the nuclear localization of p120-catenin and therefore its inhibitory effect on Kaiso transcriptional activity (Kelly et al., 2004). Phosphorylation of Ser288 is also required for p120-catenin nuclear accumulation and its ability to shuttle Kaiso to the cytoplasm, which are disrupted by the S288A mutation (Zhang et al., 2011). As *Ctnnd1*^*S288A/S288A*^ mice also did not exhibit developmental phenotypes, we conclude that Kaiso regulation is likely dispensable for development, though a role of this regulatory modality in cancer cannot be ruled out (Zhang et al., 2011; Mayerle et al., 2003; Vermeulen et al., 2012; Thoreson and Reynolds, 2002). In one out of six of the E12.5 *Ctnnd1*^*K622A,K623A/K622A,K623A*^ embryos we examined, we observed exencephaly that was similar to that observed in *Ctnnd1* neural-specific mutants (Pieters et al., 2020), which may indicate an additive effect of the loss of RhoA and Kaiso regulation by p120-catenin. However, *Ctnnd1*^*K622A,K623A/K622A,K623A*^ mice are born at a Mendelian ratio, viable, and healthy in our colony, suggesting that such an additive function for these interactions is minor in comparison with the function of p120-catenin in binding to cadherins to stabilize cell–cell adhesion.

Indeed, we find that generation of a K401M amino acid substitution in p120-catenin that specifically disrupts binding to cadherins results in a cleft lip phenotype reminiscent of epithelial loss of p120-catenin (Yu et al., 2016). Previous studies in MDCK cells showed that this mutation or complete loss of p120-catenin resulted in a dramatic change in epithelial architecture and lateral expansion of active RhoA, which could not be rescued by mutations that force stabilization of E-cadherin at the membrane. These results contributed to the interpretation that p120-catenin has important functions suppressing RhoA and actomyosin contractility at cadherin-based cell–cell contacts to regulate epithelial architecture. While that study did not identify a relevant RhoA interaction mechanism, and we did not observe any effect of disruption of the RhoA-binding domains of p120-catenin *in vivo*, we still cannot rule out that p120-catenin may regulate RhoA through some other unknown mechanism. We find, however, that the cadherin interaction domain of p120-catenin is crucial *in vivo*, leading us to favor the concept that the principal, and possibly sole, function of p120-catenin during development relates to cadherin stabilization. As MDCK cells also express P-cadherin, p120-catenin may also have an important role in its stabilization in that context, potentially explaining the discrepancy (Wu et al., 1993). Our findings are also consistent with recent studies in vascular endothelial development, where loss of p120-catenin, which is crucial for vascular integrity, can be rescued by a VE-cadherin mutation that impairs VE-cadherin endocytosis, indicating that stabilization of VE-cadherin is the main function of p120-catenin in that context (Grimsley-Myers et al., 2020).

While p120-catenin is crucial for tissue fusion in the upper lip, compound reduction of E-cadherin and P-cadherin is required to reveal their role in this process. While compensatory or synergistic roles of P-cadherin and E-cadherin have been demonstrated in the skin (Tinkle et al., 2004) and lung organoids (Hirai et al., 1989), *in vivo* interrogation of their combined function has been stymied by the genetic proximity of the *Cdh1* and *Cdh3* genes. Our gene editing strategy generates a new *Cdh1*^*lox*^; *Cdh3*^*−*^ allele for untangling this question *in vivo*. We reveal a catastrophic loss of cell adhesion and embryonic lethality in *Cdh3*^*−/−*^; *Cdh1*^*f/f*^; *Crect*^*Tg/0*^ mutant embryos prior to midface formation, supporting important and widespread collaboration of E- and P-cadherin during development. Importantly, we observed a highly specific cleft lip phenotype when E- and P-cadherin were reduced in *Cdh3*^*+/−*^; *Cdh1*^*f/f*^; *Crect*^*Tg/0*^ mutants, indicating that bolstered adherens junctions are specifically required during this epithelial fusion process. As we do not see changes in P-cadherin abundance upon loss of E-cadherin or vice versa, we do not think that one is compensating for the loss of the other. Therefore, these findings suggest that there is not a single key cadherin or a cadherin code functioning in the epithelium for fusion and instead support the idea that a critical elevated threshold of reinforced adherens junctions is required specifically at the fusion site. This cooperative role of E- and P-cadherin may parallel that seen between Nectin1 and Nectin4 during palatogenesis, where loss of both adhesion proteins resulted in a severe cleft palate while cleft palate was seen with low penetrance in single knockdowns (Lough et al., 2020).

**Figure 9.**
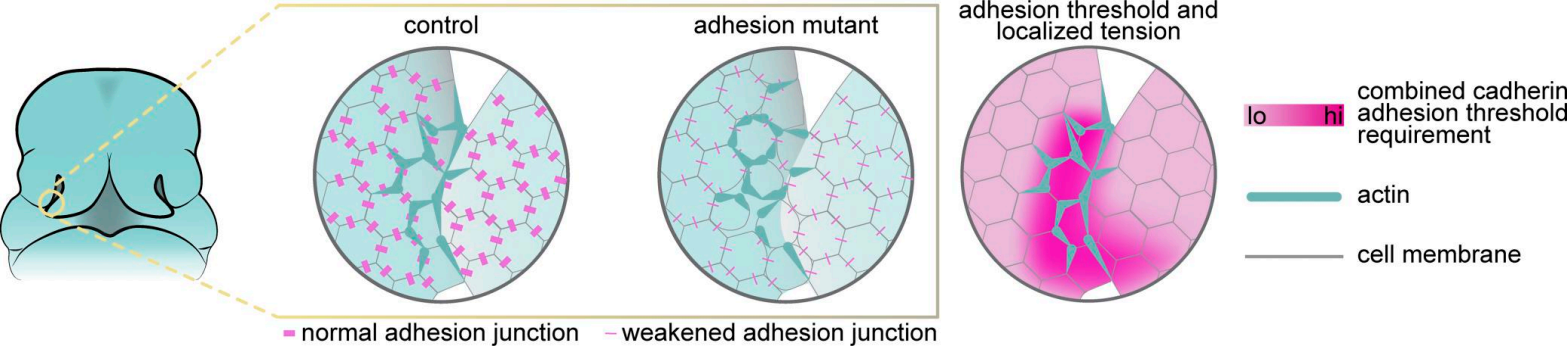
**Localized actomyosin contractility requires reinforced adherens junctions to drive upper lip fusion.** Schematic depicting our working model of the cellular mechanisms of tissue fusion in which reinforced adherens junctions are required in cells with patterned enrichment of actomyosin. Mutants with weakened adherens junctions exhibit cell rounding in cells that also exhibit high actin, suggesting actomyosin tension creates a mechanical fault zone.

Cell adhesion may play at least two distinct roles during lip fusion. First, a minimum threshold of cadherins may be required for nascent adherens complexes between cells of apposed nasal processes during initial contact. Second, high tension forces generated by actomyosin contractility needed to pull the apposing processes together may necessitate a minimum threshold of cadherin function that is higher than the surrounding non-fusing epithelium (Fig. 9). We find that this threshold requires contributions from both E-cadherin and P-cadherin, as well as reinforcement through p120-catenin stabilization. While p120-catenin is dispensable during normal *Drosophila* development, abnormalities in cell arrangement and actin organization were observed in dorsal closure tissue fusion. Notably, levels of p120-catenin junctional localization were highest in regions of the embryo undergoing morphogenetic movements (Myster et al., 2003). Together with our findings, these results suggest a conserved role of p120-catenin in strengthening cell adhesion in tissues experiencing elevated mechanical stress. We also infer, from loss of the multicellular cable patterns upon disruption of p120-catenin, that adherens junctions are crucial in propagating actomyosin tensional forces to neighboring cells during fusion. Future studies analyzing the changes in forces during lip development in mutants will be needed to understand the intricate interplay between cadherin-mediated adhesion and actomyosin tension. In addition, adherens complexes are mechanosensors that convert mechanical forces into biochemical signals (Borghi et al., 2012). A mechanotransduction role of the cadherin threshold may also be pertinent in the context of lip fusion, for example, by strengthening junctions under actomyosin contractility through catch bonds (Buckley et al., 2014). One fascinating possibility is that mechanotransduction through cadherins could result in the initiation of other cell behaviors, such as cell cycle arrest or apoptosis, which may also contribute to completion of fusion potentially in combination with other cellular behaviors (Losa et al., 2018; Qu et al., 2025, *Preprint*).

Apoptosis has often been considered as a key terminal cell behavior facilitating tissue fusion through the removal of epithelial cells. We find that regionalized cell death at the fusion junction coincides with regionally enriched F-actin. This, together with the finding that *Bak*; *Bax* mutants lacking apoptosis at the fusion site do not have cleft lip, insinuates that cell death may indeed be a consequence of increased actomyosin-generated tension and not, by itself, a predominant driver of fusion as currently thought. This is consistent with findings of abundant apoptosis that correlate with high F-actin during mammalian neural tube closure (Yamaguchi et al., 2011) and enhanced actomyosin dynamics in secondary palate fusion (Teng et al., 2022). In both cases, apoptosis has been demonstrated not to be required to complete tissue fusion (Massa et al., 2009; Teng et al., 2022). In the secondary palate, attenuated actomyosin contractility through genetic perturbation of NMII also resulted in fewer apoptotic cells. This further suggests that apoptosis is a consequence of the high actomyosin contractility that initiates tissue fusion events, though the relative functional contributions of apoptosis remain unclear (Teng et al., 2022).

In summary, we show that actomyosin contractility provides forces driving tissue fusion in the mammalian upper lip. Sensitivity of fusion to reduced cadherin-mediated cell adhesion is localized to a region of high actomyosin contractility, which manifests as a biomechanical fault point that requires p120-catenin–mediated reinforcement of adherens junctions and transduction of actomyosin-generated forces (Fig. 9). A similar mechanism may explain cleft secondary palate observed in epithelial-specific p120-catenin mouse mutants (Cox et al., 2018) and exencephaly observed in neural-specific p120-catenin mouse mutants (Pieters et al., 2020). Patients with cleft lip have previously been shown to harbor variants in *CDH1*, while variants in *CTNND1* are among the most common genetic causes of cleft lip in humans (Cox et al., 2018; Caetano da Silva et al., 2024; Diaz Perez et al., 2023). Here, we reveal novel genetic variants that further suggest that this pathway may comprise a crucial functional module that reinforces cell adhesion to withstand and transduce morphogenetic force during formation of the mammalian upper lip.

## Materials and methods

### Mouse lines

All animal experiments were performed in accordance with the protocols approved by the University of California, San Francisco Institutional Animal Care and Use Committee. The following alleles were backcrossed to, and maintained on, a coisogenic C57BL/6J genetic background: *Crect* (MGI:4887352) (Reid et al., 2011), *Bax*^*lox/lox*^ (MGI: 3589203) (Takeuchi et al., 2005), and *Bak*^−/−^ (MGI:1097161) (Lindsten et al., 2000). *Cdh1*^*f/f*^ (MGI:2389020) (Boussadia et al., 2002), *Ctnnd1*^*f/f*^ (MGI:3640772) (Elia et al., 2006), *Myh9*^*lox/lox*^ (MGI:4838521) (Jacobelli et al., 2010), and *Myh10*^*lox/lox*^ (MGI:4443039) (Ma et al., 2009) alleles were used on a mixed background.

Point mutations in *Ctnnd1* and deletion mutations in *Cdh3* were generated using the CRISPR/Cas9-based method called *i*-GONAD (Ohtsuka et al., 2018; Gurumurthy et al., 2019). For generating *Ctnnd1* variants, C57BL/6J wild-type males were crossed to CD1 wild-type females, and a 2 μl mixture comprised of the following was injected into each oviduct of female mice pregnant with E0.7 embryos and electroporated: 0.7 μg CRISPR RNA (crRNA), 0.7 μg trans-activating crRNA (tracrRNA), 2 µg donor single-stranded oligodeoxynucleotides (ssODN), and 2 ng Cas9 protein. For generating an independent *Cdh3* mutant allele, we used CD1 wild-type females crossed to C57BL/6J wild-type males. In generating a *Cdh3* allele linked to the floxed *Cdh1* allele, we used *Cdh1*^*f/f*^ females intercrossed with *Cdh1*^*f/f*^ males. For both *Cdh3* alleles, we used the following injection mixture: 0.7 μg each of two crRNAs, 1.4 μg tracrRNA, and 2 ng Cas9 protein. All crRNA, tracrRNA, ssODN, and Cas9 were acquired from IDT. Germline transmission of desired gene edits was confirmed by sequencing the progeny of candidate founders. We screened all off-target sites predicted by CHOPCHOP (Montague et al., 2014; Labun et al., 2016; Labun et al., 2019) that had fewer than three mismatches with the target sequences or were on the same chromosome with the target gene.

The *Ctnnd1*^*Y112E*^ allele includes a missense mutation that changes codon 112 from TAT (tyrosine) to GAG (glutamic acid). The *Ctnnd1*^*S288A*^ allele includes a missense mutation that changes codon 288 from AGC (serine) to GCA (alanine). No additional edits were made at these loci. The *Ctnnd1*^*K401M*^ allele includes a missense mutation changing codon 401 from AAG (lysine) to ATG (methionine) and a silent mutation in the protospacer adjacent motif sequence changing codon 405 from CGG (arginine) to CGT (arginine). The *Ctnnd1*^*K622A,K623A*^ allele includes a missense mutation that changes codons 622-623 from AAGAAG (lysines) to GCAGCA (alanines). The *Cdh3*^*Δ404*^ allele contains a 404-base pair deletion, while the *Cdh3*^*Δ398*^ allele contains a 398-base pair deletion that is linked with *Cdh1*^*flox*^. Both *Cdh3* deletions encompass exon 1, intron 1, and exon 2. Two independent founders were used to establish independent lines for each allele, except for *Cdh3*^*Δ398*^ that only had one successful founder. In all cases with two independent lines, both lines produced the same results. Detailed sequences of guide RNAs and genotyping primers are listed in Table S4.

### Live imaging

The confocal live-imaging approach was adapted from our previous work (Kim et al., 2015; Kim et al., 2017). Specifically, embryo heads were removed in phosphate-buffered saline (PBS) and transferred to culture media [20% fetal bovine serum (FBS), 100 U/ml penicillin, 100 μg/ml streptomycin, 200 μg/ml L-ascorbic acid, Dulbecco’s modified Eagle’s medium/F12 media that contain L-glutamate and HEPES and no phenol-red]. Heads were dissected leaving intact nasal processes, MXPs, mandible, and eyes. This facial tissue was then embedded in 0.6% low-melting agarose in culture media that contained SiR-Actin (1:2,000 or 0.5 μM, CY-SC001; Spirochrome) or SPY-650-FastAct_X (1:700, CY-SC500; Spirochrome). After incubation at room temperature for 15 min for the agarose to set, the tissue was incubated for another 45 min at 37°C before imaging. To inhibit actomyosin contractility, culture media–agarose mixture also contained blebbistatin (B0560; Sigma-Aldrich, 20 μM) or Y-27632 (SC-281642; Santa Cruz Biotechnology, 20 μM). Thus, treatment began 1 h prior to and continued throughout imaging. Focusing on the fusing MNP and LNP, we imaged at 10- or 20-min intervals using an inverted Zeiss Observer.Z1 spinning disk confocal microscope, a Plan-Apochromat 20× air objective with 0.8 numerical aperture, Zeiss Axiocam 506 Mono Camera, and a 37°C incubation chamber. For Y-27632 treatments and controls, samples were imaged using an inverted Zeiss LSM900 Airyscan 2 laser scanning confocal microscope, a Plan-Apochromat 40× oil objective with 1.3 numerical aperture, and a 37°C incubation chamber. Both microscopes utilize Zeiss Zen acquisition software. In experiments looking at actin in *Ctnnd1* mutants and controls, Hoechst 33342, trihydrochloride trihydrate (1:2,000, H1399; Thermo Fisher Scientific) was included in the agarose/media/SiR-Actin mixture. For these samples and *Cdh1* mutants, we acquired image stacks using a Zeiss LSM900 microscope and Plan-Apochromat 25× objective with 0.8 numerical aperture and water without continuing the time lapse. *Cdh1* mutant and respective control tissue explants were incubated in culture media containing SiR-Actin for 45 min at 37°C, then embedded in 0.6% low-melting agarose in culture media that contained SiR-Actin and calyculin A (ab141784; Abcam, 40 nM) for 15 min at room temperature prior to imaging. All images were rendered in 3D and time-lapse data made into movies using Imaris software (Oxford Instruments). As the SiR-Actin signal intensity increases over time, threshold levels were adjusted throughout the movie to circumvent signal oversaturation.

### Histology

Whole embryos were fixed in Bouin’s solution (HT101128; Sigma-Aldrich), dehydrated through an ethanol gradient, and processed through a Histo-Clear (HS-200; National Diagnostics) gradient and wax before being embedded into wax. Blocks were cut to 5-µm sections that were subsequently stained with hematoxylin and eosin. Stained sections were imaged using a Zeiss Imager.Z2, an EC Plan-Neofluar 5× air objective with numerical aperture 0.16 and Plan-Apochromat 63× oil objective with numerical aperture 1.4, an AxioCam MRc5 camera, and AxioVision software.

### Fluorescence staining

To assess cleft lip and other gross morphological changes, embryonic heads were fixed in 4% paraformaldehyde (PFA) in PBS, dehydrated through a methanol gradient, and processed in Dent’s bleach (4 parts 100% methanol, 1 part 30% hydrogen peroxide, 1 part DMSO). Samples were then rehydrated through a decreasing methanol gradient, washed in PBS + 0.1% Triton X-100, stained with DAPI (1:20,000) overnight at 4°C, and washed in PBS prior to imaging with a Zeiss Imager.Z2, an EC Plan-Neofluar 2.5× air objective with numerical aperture 0.075, an AxioCam MRm camera, and AxioVision software. *Bak*; *Bax* samples, *Myh9*; *Myh10* samples, and respective controls were imaged using a Nikon AZ100M Macro Confocal, AZ Plan Apo Air 1× lens with numerical aperture 0.1, and Nikon Elements software. Details on penetrance and unilateral/bilateral cleft can be found in Table S1.

For whole-mount immunofluorescence experiments, embryonic heads were fixed in 4% PFA in PBS, dehydrated through a methanol gradient, and processed in Dent’s bleach (4 parts 100% methanol, 1 part 30% hydrogen peroxide, 1 part DMSO). Samples were then rehydrated through a descending methanol gradient, washed in PBS, incubated in blocking solution (5% normal donkey serum, 0.1% Triton X-100 in PBS) for two hours at room temperature, and treated with primary antibodies overnight at 4°C. Following five one-hour washes in PBS, samples were treated with secondary antibodies overnight at 4°C, subjected to five 20-min washes in PBS, and stained with DAPI (1:10,000) overnight at 4°C. Lastly, samples were washed five times in PBS for 20 min each, dehydrated through a methanol gradient, and cleared through a benzyl alcohol: benzyl benzoate-in-methanol gradient (Ahnfelt-Rønne et al., 2007) before imaging with a Zeiss Observer.Z1 spinning disk confocal microscope (paired with a Plan-Apochromat 10× air objective with 0.45 numerical aperture and a C-Apochromat 40× water objective with 1.1 numerical aperture) or Zeiss LSM900 microscope (paired with a Plan-Apochromat 25× objective with 0.8 numerical aperture and water).

For cryosection immunofluorescence experiments, whole embryos were fixed in 4% PFA in PBS, dehydrated through a sucrose gradient, embedded in Tissue-Tek O.C.T. Compound, and frozen in a dry ice/ethanol bath. Blocks were cut using a CryoStar NX70 HOMP (Thermo Fisher Scientific) or a CM1900 (Leica) cryostat to 12-µm sections onto SuperFrost Plus slides (Thermo Fisher Scientific). Sections were immunostained according to standard methods with antibodies recognizing cleaved caspase-3 (1:300, 9661; Cell Signaling), E-cadherin (1:300, 13-1900; Invitrogen), p120-catenin (1:300, 610133; BD Biosciences), and P-cadherin (1:300, AF761-SP; R&D Systems). Sections were mounted in Antifade Mounting Medium and imaged with a Zeiss Imager.Z2 and an EC Plan-Neofluar 5× air objective with numerical aperture 0.16 or Zeiss LSM900 laser scanning microscope and Plan-Apochromat 25× objective with 0.8 numerical aperture and water or Plan-Apochromat 40× objective with 1.2 numerical aperture and water. For apoptosis experiments that include TUNEL technology, we utilized an *in situ* cell death detection kit (11364795910; Roche) and followed the manufacturer’s instructions.

### Quantification and statistical analysis

To quantify the intensity distribution of F-actin across the nasal processes during fusion of wild-type embryos, we used Imaris to draw a line across 3D renderings of the tissue such that the line is perpendicular to and crosses the vertex of the fusion point and spans the MNP and LNP. We then used the Surface function to create surfaces based on the F-actin signal with the following parameters in conjunction with the automated wizard: 0.5 μm surface grain size; eliminate background enabled, with largest sphere diameter of 5 μm; manual threshold of 20; morphological split enabled, with region growing estimated diameter of 3 μm; and filtered surfaces above 15.0. From this, we selected surfaces that intersected the previously drawn line and graphed the intensities of such surfaces according to their spatial positioning.

To calculate the closure between nasal processes in wild-type embryos treated with DMSO or blebbistatin, we measured the distances between the MNP and LNP in 3D using Imaris. The vertex of the fusion was visually determined, and points were placed one cell distance rostral from the vertex. In regard to anterior/posterior positioning, the two opposing points were placed on the nasal pit side of the MNP and LNP in a way that they are positioned closest together, avoiding too frontal or deep in the pit. The distance between the two points was calculated by Imaris. Statistical analysis was performed using GraphPad Prism 8, and an unpaired, two-tailed Student’s *t* test was used to determine significance.

The amount of apoptosis was quantified at two stages in *Ctnnd1*; *Crect*^*Tg/0*^ mutants and controls. Cleaved caspase-3-positive cells were manually counted through intact facial processes immunostained for cleaved caspase-3, E-cadherin, and nuclei with DAPI using ImageJ/FIJI software. At the earlier stage of 8–9ts prefusion, cleaved caspase-3-positive cells in and adjacent to the epithelium were counted. At the later stage of 17–18ts where a fusion seam exists, only cleaved caspase-3-positive cells in the epithelium were counted. Statistical analysis was performed using GraphPad Prism 8, and an unpaired, two-tailed Student’s *t* test was used to determine significance.

In *Bak*^*−/−*^; *Bax*^*f/f*^; *Crect*^*Tg/0*^ mutants, to calculate the percentage of fusion junction cells, as measured by DAPI, that is apoptotic, as measured by cleaved caspase-3, we divided the volume of DAPI signal by the volume of cleaved caspase-3 from tissue sections. Volumes were determined from surfaces created in Imaris, including only the junction epithelium. Surfaces based on the DAPI signal were created with the following parameters: 0.25 μm surface grain size; manual threshold of 1,700; morphological split enabled, with region growing estimated diameter of 1 μm; and filtered volume threshold of 496. Surfaces based on the DAPI signal were created with the following parameters in conjunction with the automated wizard: 0.25 μm surface grain size; manual threshold of 700; morphological split enabled, with region growing estimated diameter of 1 μm; and filtered volume threshold of 496. For one *Bak*^*−/−*^; *Bax*^*f/f*^; *Crect*^*Tg/0*^ mutant, we lowered the manual threshold to 300 to better capture cleaved caspase-3 signal. Statistical analysis was performed using GraphPad Prism 8, and an unpaired, two-tailed Mann–Whitney *t* test was used to determine significance.

To determine the length of F-actin–based segments in *Ctnnd1*; *Crect*^*Tg/0*^ mutants, controls, and calyculin A-treated *Cdh1*; *Crect*^*Tg/0*^ mutants and controls, we utilized the Filament function in Imaris software. Segments were traced with the following parameters in conjunction with the automated wizard: detection type of AutoPath with no loops; dendrite starting point diameter of 4.88 μm; dendrite seed point diameter of 1.50 μm; dendrite seed point threshold of 6,431 (lowered to 4,000 for a single *Ctnnd1*; *Crect*^*Tg/0*^ mutant sample, a single calyculin A-treated control sample, and a single *Cdh1*; *Crect*^*Tg/0*^ mutant sample to more faithfully detect the signal); diameter around starting point to remove seed points of 4.88 μm; and removing disconnected segment smooth width of 0.293 μm. The program wizard was trained to keep seed points associated with filaments/cables and to discard seed points associated with rounded cells or bright pixels unassociated with filaments/cables. Program-predicted seed points and segments were manually filtered to remove false-positive points or segments, though none were manually added. Statistical analysis was performed using GraphPad Prism 8, and an unpaired, two-tailed Mann–Whitney *t* test was used to determine significance.

### Analysis of whole-genome sequencing from trios with OFCs

To assess whether there were any genetic variants in *CTNND1*, *CDH1*, *CDH3*, *MYH9*, or *MYH10*, we used whole-genome sequencing data from the Gabriella Miller Kids First Pediatric Research Program (GMKF), which recruited 1,777 case-parent trios with cleft lip, cleft lip and palate, or cleft palate. Participants were recruited from the United States, Argentina, Turkey, Hungary, Spain, Colombia, and Taiwan. Participant recruitment was done at regional treatment or research centers after review and approval by each site’s institutional review board (IRB) and the IRB of the affiliated US institutions (e.g., the University of Iowa, the University of Pittsburgh, and Johns Hopkins University). Participants were assessed at the time of recruitment for additional clinical features and are largely classified as having an isolated cleft, but participants with additional clinical features consistent with a syndromic diagnosis were not excluded.

Samples from the GMKF were sequenced at the McDonnell Genome Institute (MGI), Washington University School of Medicine in St. Louis, MO, USA, or the Broad Institute in Boston, MA, USA. The variant calling and quality control methodology for 1,254 of the GMKF trios was done as part of previous studies (Bishop et al., 2020; Mukhopadhyay et al., 2020), and the quality control for the additional 523 trios was done similarly. Variants were called and aligned to the GRCh38/hg38 reference genome using the GATK pipeline (Conrad et al., 2011; McKenna et al., 2010; Van der Auwera et al., 2013). For variant-level QC, non-passing variants, variants with more than 2 Mendelian errors, variants with >5% missingness in individuals, and variants with a Hardy–Weinberg equilibrium P value <1 × 10^–7^ were removed. Additionally, calls with a genotype quality <20 or a depth <10 were set to missing and only biallelic variants were retained using VCFtools (Danecek et al., 2011). For sample-level QC, PLINK (v1.9) (Chang et al., 2015) was used to calculate identity-by-descent to confirm all family relationships and X chromosome heterozygosity to confirm the sex of all participants.

All variants were annotated to publicly available databases using ANNOVAR (v201707) (Wang et al., 2010) and Variant Effect Predictor (McLaren et al., 2016) to assess population frequency (from gnomAD v4), pathogenicity (CADD score), or effect on protein (missense, predicted loss-of-function, splicing). Noncanonical splice variants (synonymous, splice region variants) were retained if the SpliceAI score exceeded 0.5 (Jaganathan et al., 2019).

From this dataset, we extracted rare protein-altering variants (MAF <0.1% in gnomAD v4) that were present in at least one participant with a cleft. We also noted if a variant was *de novo* or inherited, and if inherited, if from a parent with a cleft. We also tested if there was a burden of DNVs in these 5 genes. To ensure that high-quality variant calls were used in analyses of DNVs, additional QC and filtering were performed. For a variant to be considered *de novo*, the variant had to be heterozygous in the proband and homozygous reference in both parents. Additionally, the variant had to have an allele balance ratio of 0.3–0.7 in the proband and <0.05 in both parents; it could not be in a known, highly repetitive region of the genome, and it had to have a minor allele frequency (MAF) <0.3% (Itai et al., 2024) in gnomAD (v3.0). Once a list of high-quality DNVs was generated, we tested for enrichment of DNVs both by class of variant (predicted loss-of-function, missense, and protein-altering) and by gene using the package DenovolyzeR in R (Samocha et al., 2014). A P value of <0.05 was considered significant. For *CTNND1*, *CDH1*, and *CDH3*, lollipop plots of variants that were *de novo*, inherited from an affected parent, or inherited from an unaffected parent were made using ProteinPaint (Zhou et al., 2016).

### Online supplemental material

Fig. S1 shows F-actin distribution and role of actomyosin contractility in wild-type embryos. Fig. S2 shows validation of epithelial-specific p120-catenin mutant. Fig. S3 shows homozygosity of p120-catenin variants. Fig. S4 shows E-cadherin and P-cadherin present in the lip epithelium throughout the fusion process. Fig. S5 shows the loss of E-cadherin does not affect F-actin enrichment in control conditions. Video 1 shows initiation of actin enrichment, related to Fig. 1. Video 2 shows expansion of actin to multicellular cables, related to Fig. 1. Video 3 shows actin zipping of nasal processes, related to Fig. 1. Video 4 shows tissue fusion proceeding in control DMSO treatment, related to Fig. 2. Video 5 shows stagnant tissue fusion with disrupted actomyosin contractility, related to Fig. 2. Video 6 shows tissue fusion in control DMSO treatment, related to Fig. S1. Video 7 shows disrupted tissue fusion with disrupted actomyosin contractility, related to Fig. S1. Table S1 shows details related to Fig. 2, 3, 4, 5, 6 and S3. Table S2 shows variants identified in *CDH1*, *CDH3*, *CTNND1*, *MYH9*, and *MYH10*, related to Fig. 7. Table S3 shows analysis of DNVs, related to Fig. 7. Table S4 shows additional methods data.

## Supplementary Material

Table S1 shows details on penetrance and unilateral/bilateral cleft.

Table S2 shows rare protein-altering variants identified in *CDH1*, *CDH3*, *CTNND1*, *MYH9*, and *MYH10*.

Table S3 shows analysis of DNVs in *CDH1*, *CDH3*, *CTNND1*, *MYH9*, and *MYH10*.

Table S4 shows gene editing and genotyping details for newly generated mouse alleles.

## Data Availability

All data associated with this study are present in the paper or the supplemental information. This paper does not report any code or informatics dataset. Any additional information required to reanalyze the data reported in this paper is available from the lead contact upon request.
