# Supplementary material for: Actomyosin contractility and a threshold of cadherin cell adhesion are required during tissue fusion
Source: J Cell Biol. 2025 Nov 13;225(1):e202503070. doi: 10.1083/jcb.202503070 (PMC12614168; doi:10.1083/jcb.202503070)
Supplement: Table S3 — shows analysis of DNVs in CDH1, CDH3, CTNND1, MYH9, and MYH10. [file jcb_202503070_tables3.docx]

**Table S3. Analysis of *de novo* variants in *CDH1*, *CDH3*, *CTNND1*, *MYH9*, *MYH10*.**

| Gene | Loss of Function  Observed | Loss of Function  Expected | Loss of Function  pValue | Protein-Altering (Miss + LoF)  Observed | Protein-Altering (Miss + LoF)  Expected | Protein-Altering (Miss + LoF)  pValue |
| --- | --- | --- | --- | --- | --- | --- |
| *CDH1* | 0 | 0 | 1 | 2 | 0.1 | **0.00526** |
| *CTNND1* | 1 | 0 | 0.0135 | 2 | 0.1 | **0.00695** |
| *MYH10* | 1 | 0 | 0.0214 | 1 | 0.2 | 0.221 |
| *CDH3* | 0 | 0 | 1 | 1 | 0.1 | 0.0996 |
| All Genes | 2 | 0.1 | **0.00323** | 6 | 0.9 | 0.000288 |
| *CDH3, MYH9, MYH10* | 1 | 0 | 0.0322 | 2 | 0.4 | 0.049 |
